# Bioactivity-Guided Optimization of 3-Deoxyanthocyanidin Extraction and Seasonal Mapping of Four Morphotypes of *Fridericia chica* (Bonpl.) L.G. Lohmann

**DOI:** 10.3390/ijms27188193

**Published:** 2026-09-15

**Authors:** Paulo Victor R. Souza, Ygor Jessé Ramos, João Victor Silva-Silva, Maria do Socorro S. Chagas, Adélia Viviane de Luna, Katia S. Calabrese, Fernando Almeida-Souza, Davyson L. Moreira, Carla J. Moragas-Tellis, Maria D. Behrens

**Affiliations:** 1 Laboratory of Natural Products for Public Health, Pharmaceutical Technology Institute, Farmanguinhos, Oswaldo Cruz Foundation, Rio de Janeiro 21040-900, RJ, Brazil; maria.chagas@fiocruz.br (M.d.S.S.C.); adelia.luna@fiocruz.br (A.V.d.L.); carla.tellis@fiocruz.br (C.J.M.-T.); maria.behrens@fiocruz.br (M.D.B.); 2 Farmácia da Terra Laboratory, School of Pharmacy, Federal University of Bahia, Salvador 40170-115, BA, Brazil; ygor.jesse@ufba.br; 3 Institute of Mathematics and Computer Sciences, University of São Paulo, São Carlos 13566-590, SP, Brazil; jvictorslvs@gmail.com; 4 Laboratory of Protozoology, Oswaldo Cruz Institute, Oswaldo Cruz Foundation, Rio de Janeiro 21040-900, RJ, Brazil; calabrese@ioc.fiocruz.br (K.S.C.); fernandoalsouza@gmail.com (F.A.-S.); 5 Postgraduate Program in Animal Science, State University of Maranhão, São Luis 65055-310, MA, Brazil; 6 Natural Products and Biochemistry Laboratory, Rio de Janeiro Botanical Garden Research Institute, Rio de Janeiro 22460-030, RJ, Brazil; davysonmoreira@jbrj.gov.br

**Keywords:** *Fridericia chica*, *Leishmania amazonensis*, 3-deoxyanthocyanidins, carajurin, DoE, UAE, HPLC analysis

## Abstract

Ultrasound-assisted extraction (UAE) was combined with a design of experiments (DoE) to optimize the recovery of 3-deoxyanthocyanidins (3D-anths) from *Fridericia chica* leaves using in vitro antileishmanial activity against *Leishmania amazonensis* promastigotes as a bioactivity-guided response. A 3 × 3 factorial design evaluated extraction time (15–45 min) and ethanol concentration (70–100%), followed by HPLC-DAD quantification, cytotoxicity testing, and seasonal mapping of four morphotypes over 21 months. Ethanol concentration was the main determinant of extraction performance, and 100% ethanol produced the most active extracts. Assay C (15 min, 100% ethanol) showed the highest selectivity index (18.5); however, assay F (30 min, 100% ethanol) was selected through a supervised multi-criteria assessment because it combined favorable activity with the highest total 3D-anth content (12.58%) and carajurin recovery (10.37%). Chromatographic profiles remained qualitatively stable within morphotypes, although quantitative variation occurred among months. Carajurin predominated in AC1 and AC2, whereas 3′-hydroxy-carajurone predominated in AC3 and AC4. Most associations between the evaluated meteorological variables and 3D-anths content was not statistically significant; moderate negative correlations with relative humidity were detected for selected metabolites in AC1, AC2, and AC4. Hierarchical clustering produced four clusters consistent with the predefined morphotype classification in this dataset. These findings support UAE-DoE as a strategy for chemical standardization of *F. chica* extracts.

## 1. Introduction

Leishmaniasis is among the world’s most neglected tropical diseases and is endemic in approximately 100 countries. More than 1 billion people live in areas at risk of infection, and approximately 1 million new cases are reported annually [1]. Despite increased attention in recent years, this protozoan disease remains a major global health challenge. Limited therapeutic options, drug resistance, substantial toxicity, and high treatment costs hinder disease control [2].

Accordingly, there is an urgent need to discover or develop more effective drugs that can replace or complement current antileishmanial chemotherapy. Owing to their broad chemical diversity, natural products have contributed to the identification of candidate compounds with promising antiprotozoal activity. Medicinal plants may therefore provide useful sources of safer and more effective therapeutic leads against leishmaniasis [3,4,5].

*Fridericia chica* (Bonpl.) L.G. Lohmann (syn. *Arrabidaea chica* (Humb. & Bonpl.) B. Verlot), commonly known in Brazil as crajiru or pariri, is native to the Amazon rainforest and belongs to the family Bignoniaceae, which comprises approximately 120 genera and 860 species [5,6,7]. The species is traditionally used medicinally, including leaf tea for skin inflammation and mycoses. Indigenous Amazonian communities also use leaf decoctions to cleanse wounds and ulcers, support healing, and treat fungal infections and herpes [8].

*F. chica* is characterized by the presence of 3-deoxyanthocyanidins (3D-anths). Although these compounds share a biosynthetic origin with other flavonoids, anthocyanidins have distinct structural and spectroscopic properties. Continuous delocalization of π electrons across the conjugated ring system results in absorption in the visible region, generally between 480 and 540 nm. For the 3D-anths analyzed in this study, detection was performed at 480 nm [9,10,11].

**Figure 1:**
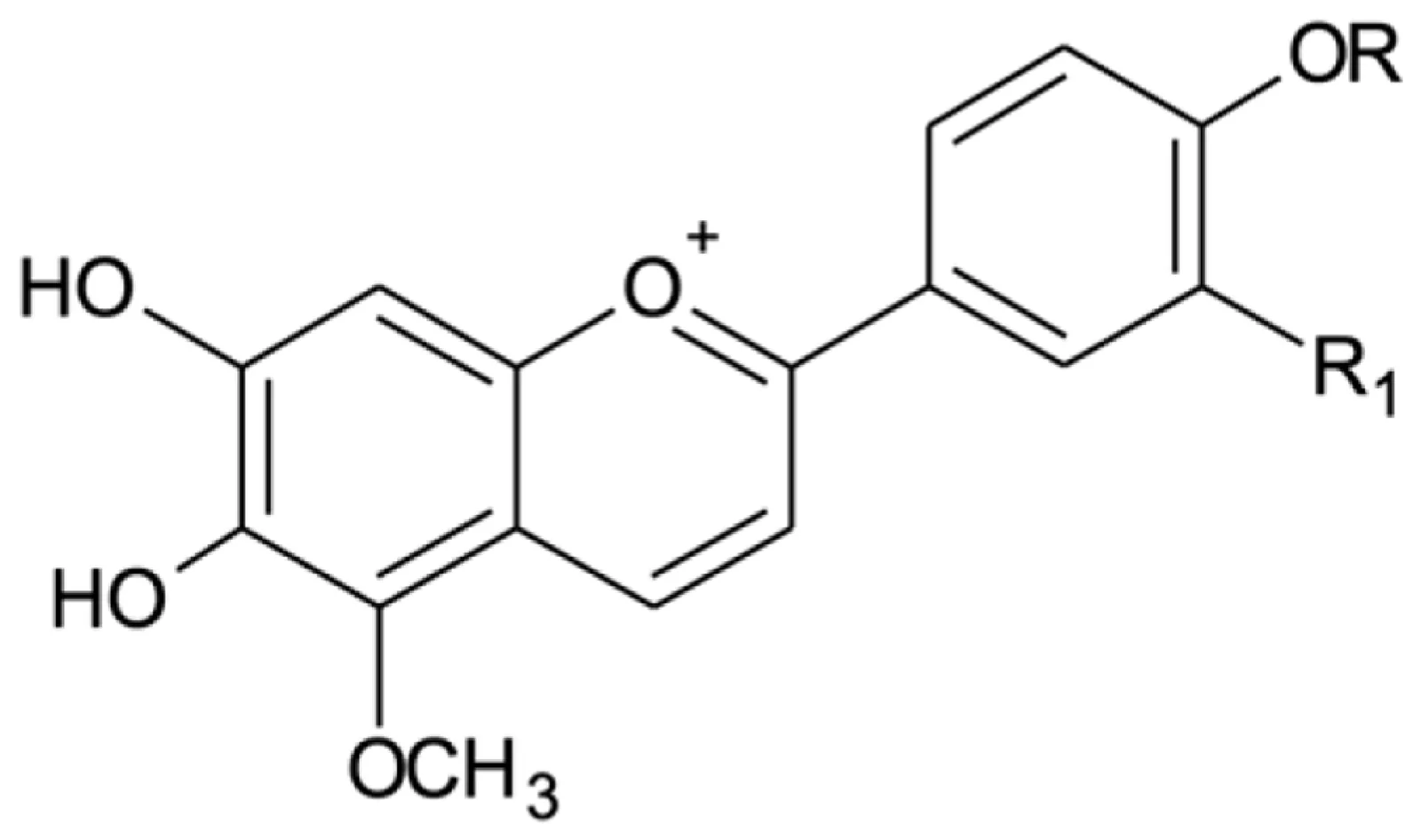
Chemical structures of the 3D-anths described for *Fridericia chica*: 3′-hydroxy-carajurone (R_1_ = OH; R = H); carajurone (R_1_ = H; R = H); 3′-hydroxy-carajurin (R_1_ = OH; R = CH_3_); and carajurin (R_1_ = H; R = CH_3_).

The extraction, isolation, and chemical characterization of four 3D-anths from *F. chica* have been previously described [6,12,13]: 6,7,3′,4′-tetrahydroxy-5-methoxyflavylium (3′-hydroxy-carajurone); 6,7,4′-trihydroxy-5-methoxyflavylium (carajurone); 6,7,3′-trihydroxy-5,4′-dimethoxyflavylium (3′-hydroxy-carajurin); and 6,7-dihydroxy-5,4′-dimethoxyflavylium (carajurin) (Figure 1). Carajurin is the most extensively studied 3D-anths from *F. chica* and has not yet been reported from another plant species [6,14].

Several pharmacological studies have reported cytotoxic [15,16], genotoxic [17], wound-healing [15,18,19,20], antimicrobial [16,21], anti-inflammatory [22,23,24], and antioxidant activities [25,26,27] for *F. chica*, among other effects. Antileishmanial activity has also been reported [15,28] and has been investigated extensively by our research group. Initial studies showed that isolated flavones and flavone-rich fractions were active against intracellular amastigotes [4]. Moreover, variation in the 3D-anths profiles of extracts from four *F. chica* morphotypes affected antileishmanial activity [6], suggesting a possible association with carajurin. Based on combined experimental and literature evidence, carajurin was subsequently proposed as a biological marker of antileishmanial activity in *F. chica* [29]. Further mechanistic studies, including structure–activity relationship analyses, suggested that carajurin targets parasite mitochondria and induces apoptosis-like cell death in *Leishmania amazonensis* promastigotes [5].

Because conventional plant extraction methods, such as maceration and Soxhlet extraction, are time-consuming and require large volumes of solvent, emerging extraction technologies have been developed and increasingly adopted [30,31]. Among these, ultrasound-assisted extraction (UAE) is widely used as an efficient, cost-effective, and comparatively environmentally favorable technique for recovering bioactive compounds from natural products [32,33,34].

Design of experiments (DoE) provides a systematic framework for evaluating relationships among multiple factors and their effects on process responses, unlike traditional one-factor-at-a-time approaches. Process variables may interact, such that the effect of one factor depends on the level of another. These interactions can be identified through experimental designs that reduce the number of runs while maximizing the information obtained [35,36]. DoE is also consistent with Green Chemistry principles because it can reduce chemical and resource consumption [37].

In the present study, a DoE approach was used to evaluate how ethanol concentration in the extraction solution and UAE time affected *F. chica* leaf-extract yield, 3D-anths content, and antileishmanial activity. After optimization, seasonal variation in the four *F. chica* morphotypes was assessed over 21 months using multivariate analysis of 3D-anths composition and correlation analyses between 3D-anths content and abiotic factors.

## 2. Results and Discussion

### 2.1. Effects of Extraction Conditions on Process Optimization

The dependent variables—extract yield, 3D-anths content, and biological responses—are presented in Table 1. The extract yield ranged from 15.59% to 32.19%, depending on the combination of independent variables used in each assay. The content of 3′-hydroxy-carajurone ranged from 0.11% to 0.85%, that of carajurone from 0.17% to 1.36%, and that of carajurin from 0.83% to 10.37%. Carajurin was therefore the most abundant 3D-anths in the evaluated extract. 3′-Hydroxy-carajurin was not detected in the AC2 morphotype used in the experimental design. At 72 h, the lowest IC_50_ values were obtained in assays C (6.09 ± 1.10 µg/mL), F (7.22 ± 1.09 µg/mL), and I (9.87 ± 1.18 µg/mL), all performed with 100% ethanol. The highest CC_50_ values, indicating the lowest cytotoxicity, were observed in assays A (655.10 ± 1.24 µg/mL) and B (569.20 ± 1.22 µg/mL), followed by assay H (403.00 ± 1.14 µg/mL). The selectivity index (SI; CC_50_/IC_50_) was highest for assays C (18.5) and F (7.5), which also showed strong inhibitory activity.

Analysis of variance (ANOVA; Table 2) showed that carajurone and carajurin content was significantly affected by the linear and quadratic terms of *X*1 and *X*2 (*p* < 0.05). For 3′-hydroxy-carajurone, the quadratic term of *X*1 and the linear term of *X*2 were significant. The linear term *X*2 also significantly affected extract yield, IC_50_, and SI. No statistically significant model terms were identified for CC_50_ (*p* > 0.05). The linear interaction between *X*1 and *X*2 was not significant for any dependent variable.

The data were subjected to multiple regression analysis, from which fitted models were generated for the seven dependent variables [38]. Table 3 presents the resulting equations describing the empirical relationships between each response and the independent variables. The coefficients of determination (*R*^2^) ranged from 0.890 to 0.994. Although these values indicate that a large proportion of response variability was accounted for by the fitted equations, the CC_50_ model was not statistically significant and should be interpreted cautiously. The models for 3D-anths content showed *R*^2^ values greater than 0.98.

**Figure 2:**
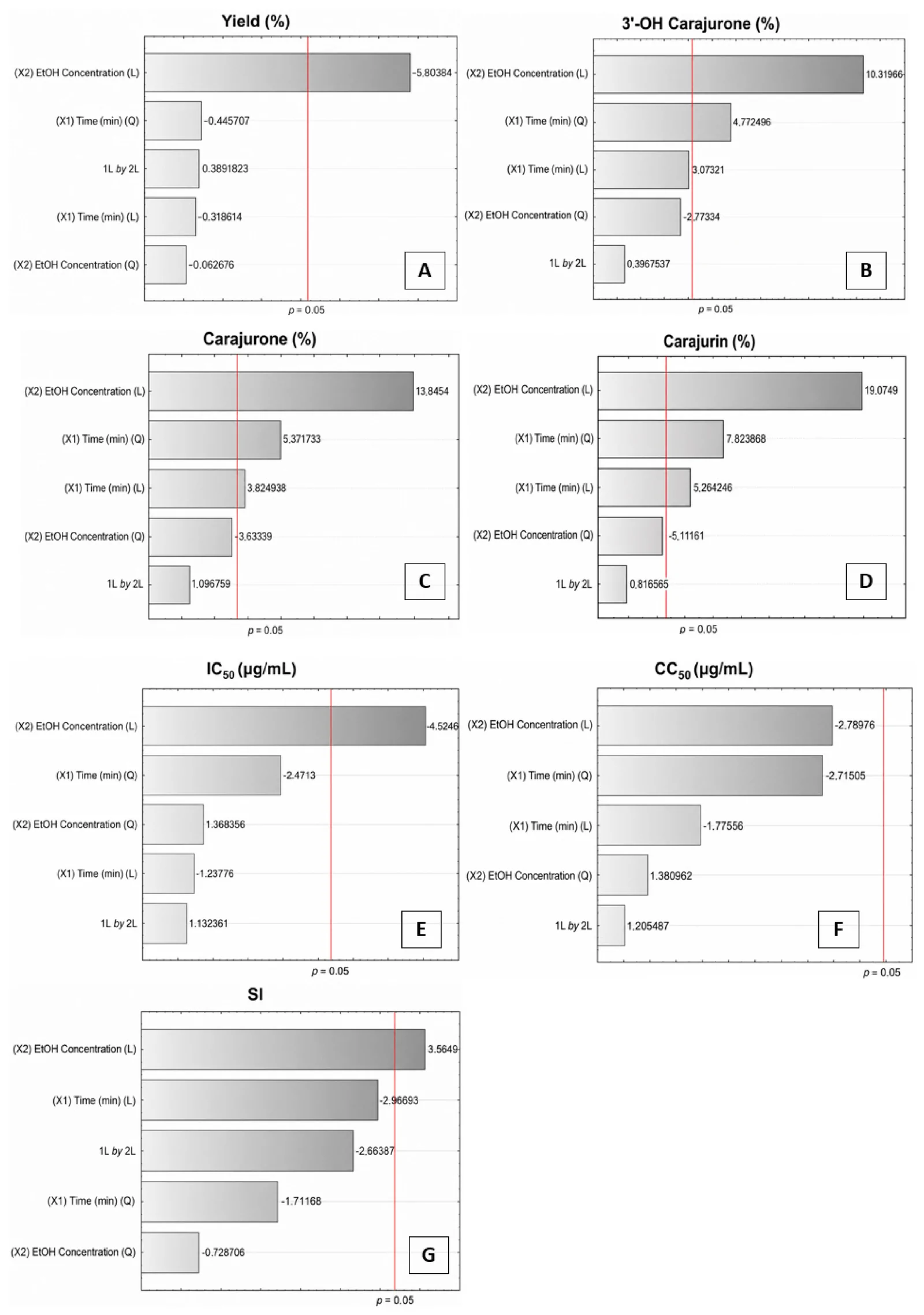
Pareto charts generated from standardized effect estimates of the dependent variables as functions of the independent factors (L = linear; Q = quadratic; 1L *by* 2L = linear interaction between the independent variables). (**A**) Yield; (**B**) 3′-hydroxy-carajurone; (**C**) carajurone; (**D**) carajurin; (**E**) IC_50_; (**F**) CC_50_; (**G**) SI.

The Pareto charts in Figure 2 show the standardized effects of the independent variables and should be interpreted together with the term-specific ANOVA results in Table 2. The linear and quadratic terms of *X*1 were significant for carajurone and carajurin, whereas the linear *X*2 term was significant for both compounds and the quadratic *X*2 term also reached significance in the ANOVA (Table 2). Ethanol concentration (*X*2) was the most influential factor for most dependent variables. For extract yield (Figure 2A) and IC_50_ (Figure 2E), the linear *X*2 term had a significant negative effect (*p* < 0.05), indicating that higher ethanol concentrations reduced both responses. For 3′-hydroxy-carajurone, the quadratic *X*1 and linear *X*2 terms were significant. No significant effects were detected for CC_50_ (Figure 2F). Minor visual differences between the Pareto threshold display and Table 2 reflect rounding of standardized effects; inferential conclusions were based on the ANOVA *p*-values reported in Table 2.

**Figure 3:**
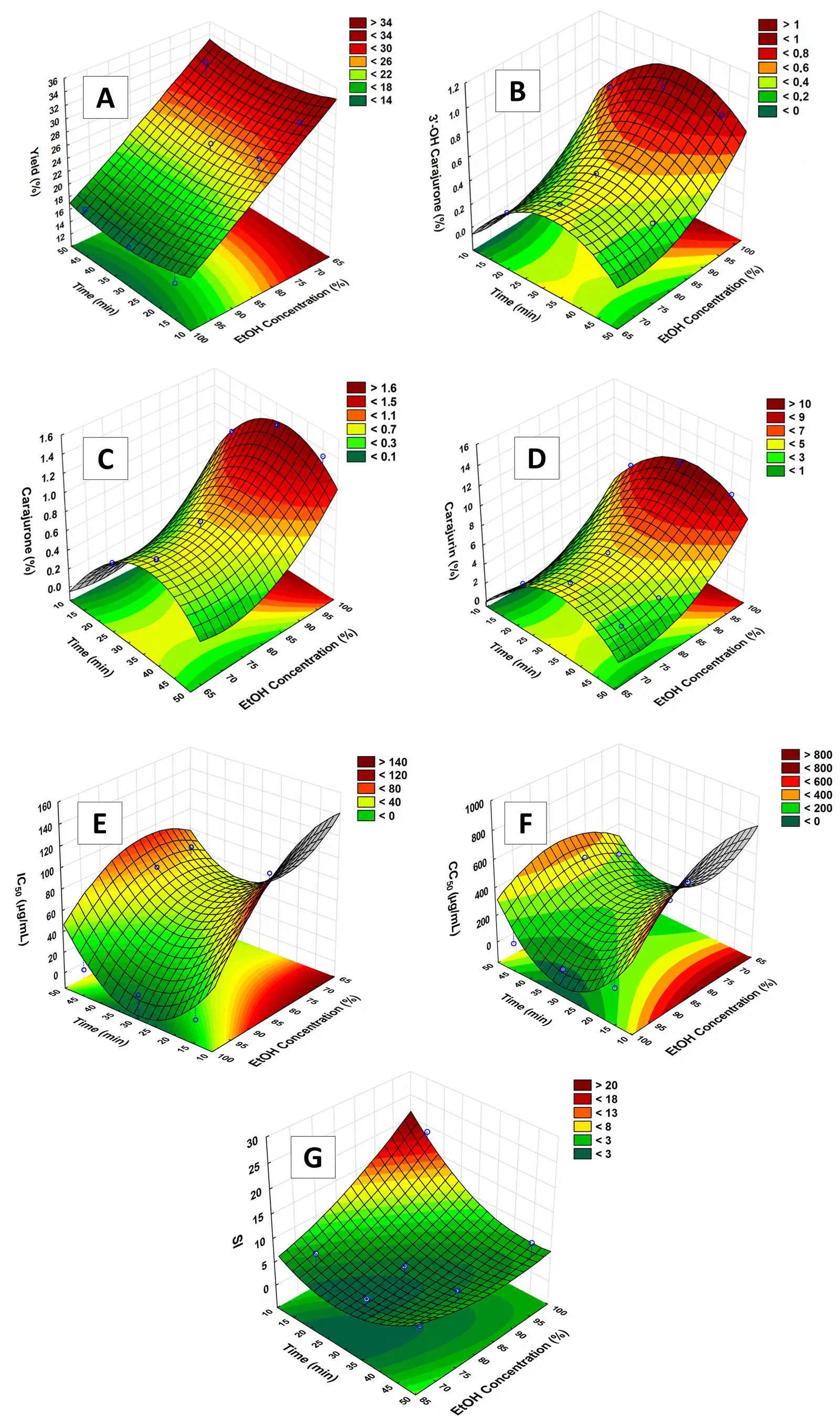
Response surface plots generated as functions of the analyzed factors (independent variables): (**A**) yield; (**B**) 3′-hydroxy-carajurone; (**C**) carajurone; (**D**) carajurin; (**E**) IC_50_; (**F**) CC_50_; (**G**) SI.

The response surface plots derived from each fitted mathematical model are shown in Figure 3. These plots represent the dependent variables on the *Z*-axis as functions of factors *X*1 and *X*2. The blue circles are the graphical representation of each of the conditions for tests A–I conducted in this study.

Figure 3A shows that extraction time had little effect on overall extract yield. In contrast, ethanol concentration exerted a negative linear effect, with increasing ethanol concentration reducing the overall yield. This finding confirms the influence of ethanol concentration identified in the preceding statistical analyses. The extraction process may have reached equilibrium at all evaluated times, a common pattern in which extraction efficiency initially increases and then reaches a maximum [39].

The response surfaces for 3D-anths content (Figure 3B–D) were convex, with the lowest responses at the extremes of *X*1 and the most favorable responses at the intermediate extraction time (30 min). Unlike overall extract yield, ethanol concentration had a positive effect on 3D-anths content, and carajurin reached the highest concentration among the three compounds evaluated. These data suggest that extraction time may affect process selectivity, possibly because of differences in the solubilization kinetics of the anthocyanidins or time-dependent changes in their physicochemical behavior during UAE [33,40].

The response surfaces for the biological assays (Figure 3E,F) were concave, with the highest numerical responses at the shortest and longest extraction times. For IC_50_, however, lower values indicate greater activity, and the most favorable response occurred at intermediate conditions. The SI response (Figure 3G) was highest at the shortest extraction time and greatest ethanol concentration.

The results indicate that ethanol concentration (*X*2) played a major role in most responses. The highest overall extract yields were obtained at the lowest ethanol concentrations. This finding may be attributed to the higher water content, which favors extraction of several classes of polar compounds through hydrogen-bonding interactions and increased solubility in the aqueous phase [41].

Conversely, ethanol, which is generally considered a comparatively safe organic solvent, can favor the extraction of less polar compounds [42]. This explains the higher 3D-anth content in assays performed with 100% ethanol, because anthocyanidins are the aglycone forms of anthocyanins and are less soluble in highly aqueous media. Nevertheless, anthocyanidin solubility may remain limited even in less polar solvents [9]. Thus, assays C, F, and I, which used 100% ethanol, provided the most favorable results for the target 3D-anths, as described above.

Phase-equilibrium behavior may also influence these results. Changes in extraction-medium temperature caused by ultrasonic processing, together with changes in solvent composition, can alter solute activity coefficients and solubility [33,43,44]. Increasing the ethanol proportion may preferentially solubilize other compounds and alter concentration gradients and mass transfer. However, changes in solution composition may also affect the equilibrium behavior of the target compounds and reduce their recovery. Analogous behavior was reported for the extraction of flavonoids from *Flaveria bidentis* (L.) Kuntze (Asteraceae) [44].

The IC_50_ and SI responses were also most favorable in assays C, F, and I, coinciding with the conditions that yielded the highest 3D-anths content. However, these assays also produced the lowest CC_50_ values. Nevertheless, the SI, calculated as the CC_50_/IC_50_ ratio, remained highest for assays C and F (SI_C_ = 18.5; SI_F_ = 7.5).

Inspection of the 3D-anths structures (Figure 1) shows that carajurin has the greatest degree of methoxylation, which may contribute to its stronger antileishmanial activity [29]. In contrast, 3′-hydroxy-carajurone and carajurone are more hydroxylated analogues and were associated with greater cytotoxicity in previous studies by our research group [4]. These findings were relevant when comparing assays C (15 min; 100% ethanol) and F (30 min; 100% ethanol). Assay C produced the highest SI (18.5), whereas assay F produced the second-highest SI (7.5) but higher individual and total 3D-anths content. The lower SI of assay F may be related to its higher content of 3′-hydroxy-carajurone and carajurone, which could have increased cytotoxicity [4].

To examine the relationship between chemical composition and antileishmanial activity within the experimental design, exploratory Pearson correlations were calculated between individual 3D-anths content in the nine DoE conditions and their corresponding IC_50_ values. Strong negative correlations were observed for 3′-hydroxy-carajurone (r = −0.965, *p* < 0.001), carajurone (r = −0.943, *p* < 0.001), and carajurin (r = −0.948, *p* < 0.001), indicating that higher concentrations of these metabolites were associated with lower IC_50_ values within this dataset. Because the analysis included only nine conditions and multiple related metabolites, these correlations are considered exploratory rather than evidence of independent causal effects. Experimental confirmation using three independently prepared batches showed agreement between predicted and observed responses for extraction yield and individual and total 3D-anths content, with relative errors of 1.1–9.1% and low interbatch variability. The biological responses fell within the corresponding 95% prediction intervals but showed larger relative deviations and were interpreted cautiously. Complete validation results, including model-predicted and observed values, standard deviations, relative errors, and the lower and upper limits of the 95% prediction intervals, are provided in Supplementary Table S1. No formal automated desirability function was applied. Instead, the optimum was selected through a supervised multi-criteria assessment in which recovery of carajurin and total 3D-anths was prioritized while retaining favorable biological activity. Although assay C had a higher SI (18.5 vs. 7.5) and a numerically lower IC_50_ than assay F, the IC_50_ difference was not statistically significant. Assay F provided 38.8% greater carajurin recovery (10.37% vs. 7.47%) and 37.3% greater total 3D-anth content (12.58% vs. 9.16%). Assay F (30 min; 100% ethanol) was therefore selected because it provided the most favorable balance between biological activity, active-marker enrichment, standardization, and potential process scalability within the evaluated domain.

### 2.2. Seasonal Variation in 3D-Anths Content of Fridericia chica

#### 2.2.1. Chromatographic Profiles of 3D-Anths in *Fridericia chica* Extracts by HPLC-DAD

**Figure 4:**
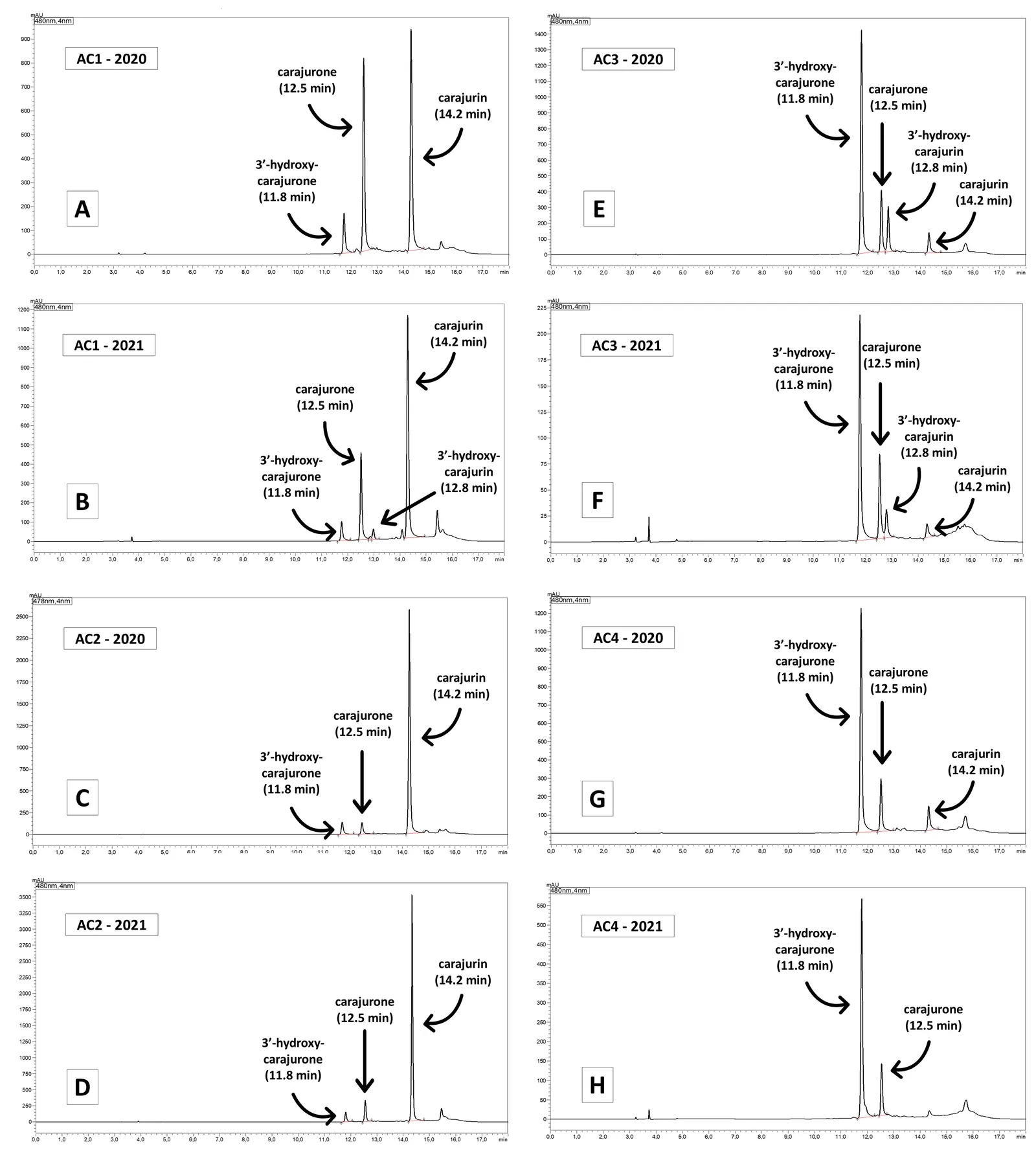
Chromatographic profiles of crude extracts obtained by ultrasound-assisted extraction from samples of the four *Fridericia chica* morphotypes collected in October 2020 and October 2021: (**A**) AC1-2020; (**B**) AC1-2021; (**C**) AC2-2020; (**D**) AC2-2021; (**E**) AC3-2020; (**F**) AC3-2021; (**G**) AC4-2020; (**H**) AC4-2021.

After optimization of the UAE process, crude ethanolic extracts of the four morphotypes collected in October 2020 and October 2021 were analyzed to compare their chromatographic profiles (Figure 4). The HPLC-DAD method had been previously developed and validated by our research group [11], and compound identification at 480 nm followed the protocol described in [6]. The 3D-anths eluted in the following order: 3′-hydroxy-carajurone (RT = 11.8 min), carajurone (RT = 12.5 min), 3′-hydroxy-carajurin (RT = 12.8 min), and carajurin (RT = 14.2 min).

In morphotype AC1, carajurin was the major compound in both years, followed by 3′-hydroxy-carajurone and carajurone, with these compounds occurring at a lower relative abundance in 2020 (Figure 4A). Only trace amounts of 3′-hydroxy-carajurin were detected in the 2021 extracts (Figure 4B).

In morphotype AC2, carajurin was also the predominant compound in both years, with a markedly higher relative abundance than 3′-hydroxy-carajurone and carajurone, as indicated by the chromatographic peak intensities (Figure 4C,D).

Morphotype AC3 (Figure 4E,F) was the only morphotype in which all four compounds were detected throughout the collection period. 3′-Hydroxy-carajurone predominated, followed by the other three compounds in their chromatographic elution order. The profiles were similar in the two years, although peak intensities were generally higher in 2020 (Figure 4E).

In morphotype AC4 (Figure 4G,H), 3′-hydroxy-carajurone was again predominant, followed by carajurone at lower concentrations. Only these two compounds were detected in 2021 (Figure 4H). 3′-Hydroxy-carajurin and carajurin were detected only at low levels in samples from 2020, at concentrations close to the analytical limits of detection and quantification (Figure 4G).

#### 2.2.2. Seasonal Quantification, Climatic Associations, and Putative Biosynthetic Relationships of 3D-Anths in *F. chica*

**Figure 5:**
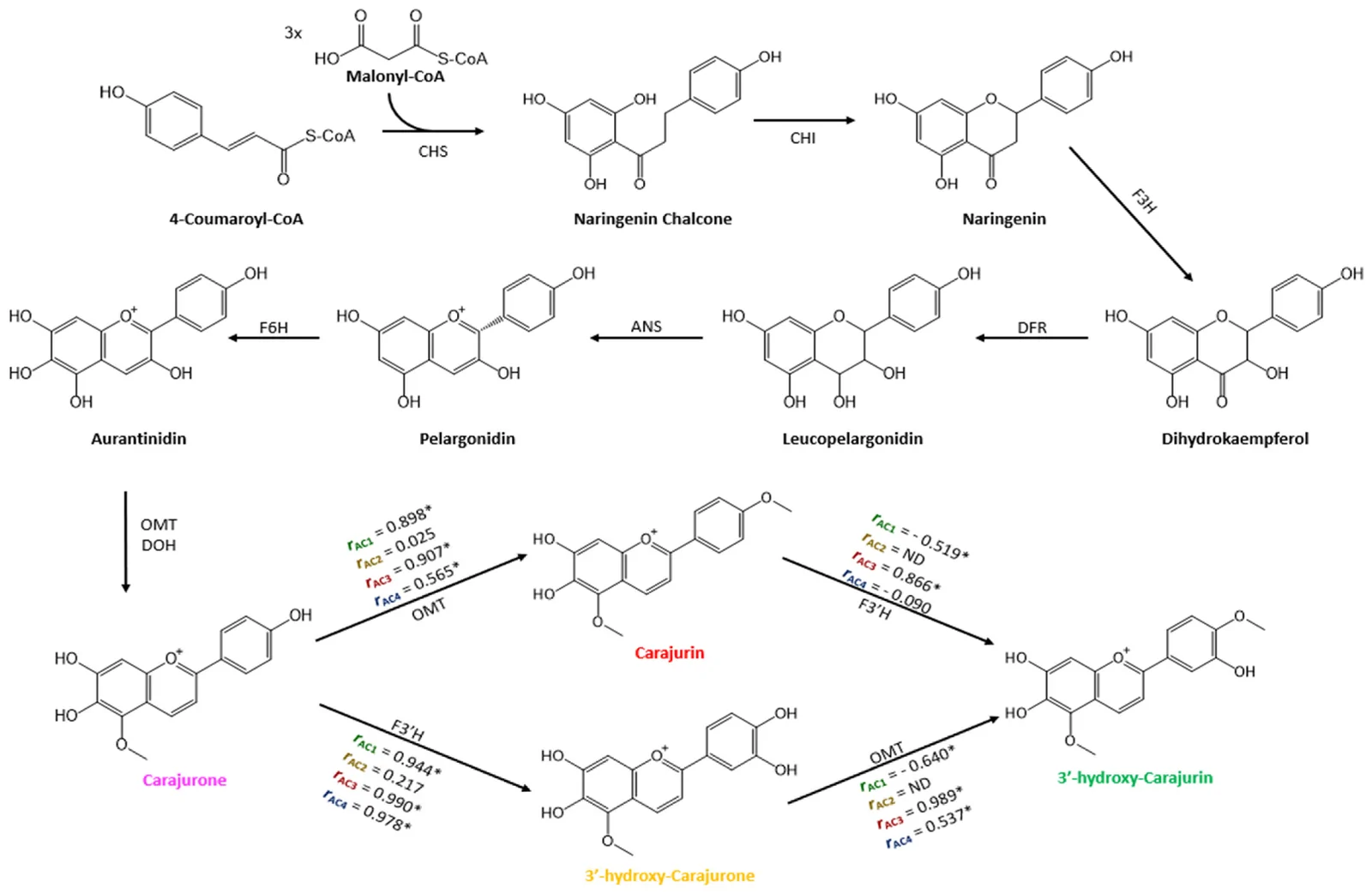
Putative biosynthetic relationships and correlation coefficients (*r*) for 3D-anths from *Fridericia chica*. CHS = chalcone synthase; CHI = chalcone isomerase; F3H = flavonoid 3-hydroxylase; F3′H = flavonoid 3′-hydroxylase; F6H = flavonoid 6-hydroxylase; DFR = dihydroflavonol reductase; ANS = anthocyanidin synthase; OMT = *O*-methyltransferase; DOH = putative dehydroxylation step; ***r*****_ACX_** = correlation coefficient between compounds for morphotype ACX; * *p* < 0.05; ND = not detected. Correlations do not establish reaction directionality or enzyme identity.

Using a calibration curve generated with an isolated carajurin standard (*R*^2^ = 0.997) [6], all 3D-anths were quantified and reported as carajurin equivalents. This approach was adopted because purified analytical standards were unavailable for the other three structurally related 3D-anths. Accordingly, their values are semi-quantitative and may be affected by compound-specific differences in molar absorptivity and detector response at 480 nm. Based on these results and previous literature [45,46,47], tentative biosynthetic relationships among the detected 3D-anths were proposed (Figure 5). The early flavonoid pathway begins with condensation of *p*-coumaroyl-CoA, derived from phenylalanine through the shikimate pathway, with three malonyl-CoA units derived from the acetate pathway. Chalcone synthase (CHS) catalyzes the formation of naringenin chalcone, which chalcone isomerase (CHI) converts to naringenin. Subsequent transformations leading to the 3D-anths detected in *F. chica* were inferred from compound occurrence and correlation patterns. Because no enzyme-activity, gene-expression, or isotope-tracing data were generated, Figure 5 represents a working hypothesis rather than a confirmed biosynthetic pathway. The correlations are compatible with possible hydroxylation, O-methylation, and dehydroxylation relationships, but do not establish reaction directionality, precursor–product relationships, or enzyme identity.

**Figure 6:**
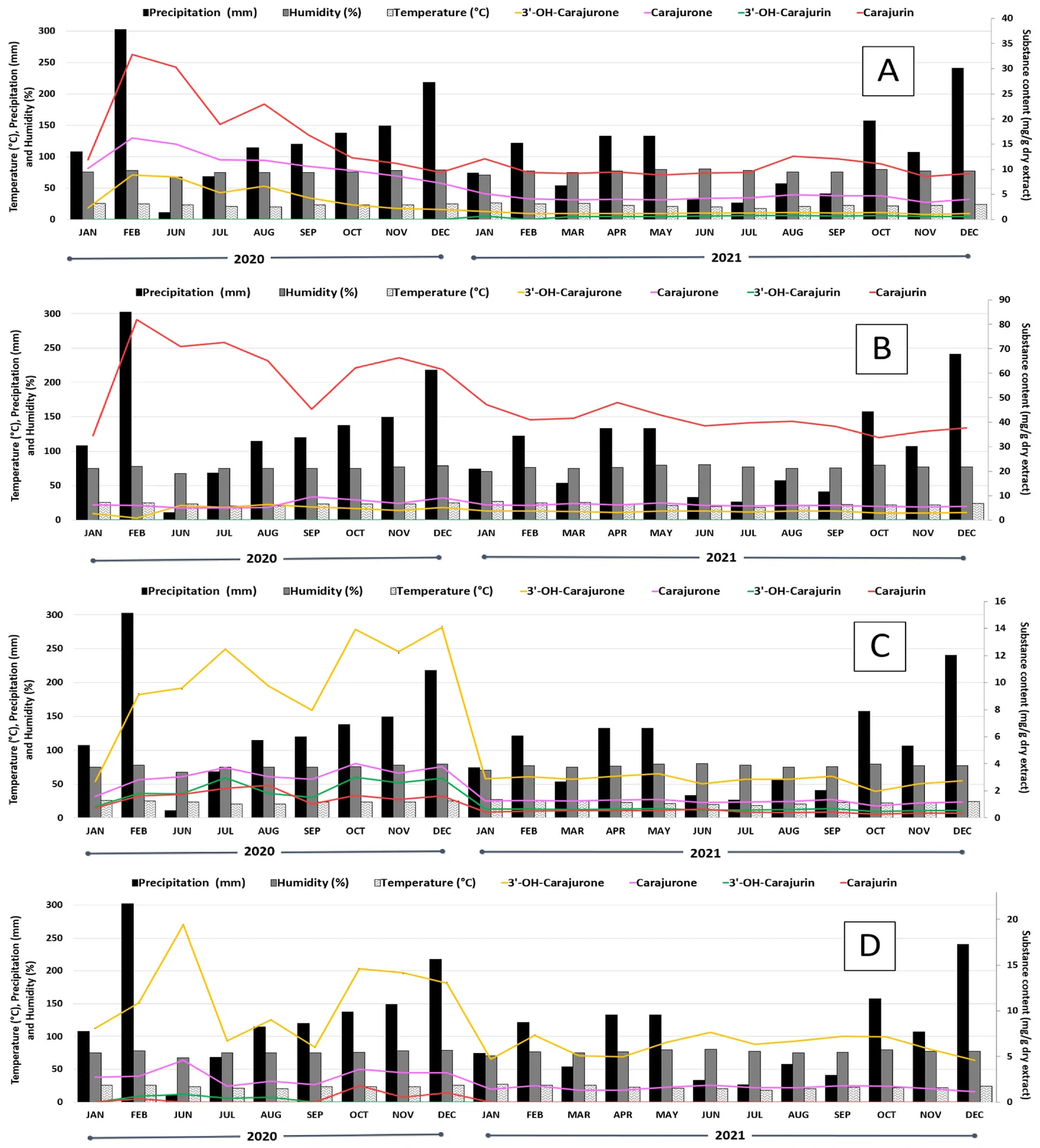
Climatological data and seasonal variation in 3D-anths in crude ethanolic extracts of morphotypes AC1 (**A**), AC2 (**B**), AC3 (**C**), and AC4 (**D**) of *Fridericia chica*, collected monthly during 2020 and 2021. OH = hydroxyl group.

Overall, the climatic data shown in Figure 6 suggested that periods of increased accumulated precipitation preceded reductions in 3D-anths content, and the first collection year was rainier than the second. However, no statistically significant correlations were detected between accumulated precipitation and the evaluated compounds (*p* > 0.05). Correlations between 3D-anths content and the climatic variables are presented in Supplementary Materials.

For morphotype AC1 (Table 4), carajurin showed the highest content in both collection years, ranging from 8.52 to 32.83 mg/g (*p* < 0.05). 3′-Hydroxy-carajurone (1.02–8.84 mg/g) and carajurone (3.41–16.20 mg/g) were also detected, whereas 3′-hydroxy-carajurin was detected only in 2021 and at much lower content (0.56–0.82 mg/g). Carajurin content in 2020 changed significantly (*p* < 0.05) among months and relative to the other compounds, except in January (11.86 mg/g) and December (9.37 mg/g). Overall, content was higher in 2020 than in 2021, except for 3′-hydroxy-carajurin, which was detected only in 2021. Carajurin content in 2021 and carajurone content in 2020 were statistically similar (*p* > 0.05).

Within morphotype AC1, significant positive correlations were observed among several 3D-anths over the two collection years. Higher 3′-hydroxy-carajurone content was associated with higher carajurone (*r* = 0.944; *p* < 0.001) and carajurin content (*r* = 0.988; *p* < 0.001). In contrast, correlations involving 3′-hydroxy-carajurin were negative. These associations are compatible with differences in hydroxylation and *O*-methylation patterns but do not demonstrate biosynthetic directionality.

The AC1 profile (Figure 6A) showed that the highest total 3D-anths content, recorded in February 2020, coincided with the greatest accumulated rainfall over the two collection years (302.4 mm). June 2020 also showed one of the highest levels of 3D-anths content despite being the driest month (11 mm). During 2020, 3D-anths content generally declined after February, with a small increase in August. During 2021, the content remained comparatively stable.

No strong correlations were detected between 3D-anths content and the climatic variables. The only statistically significant association was a moderate negative correlation between carajurin and relative humidity (*r* = −0.461; *p* < 0.05) (Supplementary Table S2). This isolated exploratory association indicates that the observed chemical variation was not consistently explained by the measured environmental variables and may reflect unmeasured environmental, developmental, or physiological factors.

In morphotype AC2 (Table 5), carajurin was the major compound, with content ranging from 33.57 to 81.90 mg/g and significantly exceeding that of 3′-hydroxy-carajurone (2.63–6.40 mg/g) and carajurone (4.91–9.54 mg/g) in both collection years (*p* < 0.05). 3′-Hydroxy-carajurin was not detected. Carajurin content differed significantly among most months, except in June (38.50 mg/g) and September 2021 (38.28 mg/g), which were statistically similar (*p* > 0.05). The high carajurin content also distinguished AC2 from the other morphotypes (*p* < 0.05).

As in AC1, carajurin content in AC2 was consistently higher than in the other 3D-anths, although the difference was approximately tenfold greater (Figure 6B). The other two detected compounds remained comparatively stable from 2020 to 2021. Carajurin reached its highest content in February (81.90 mg/g) and July 2020 (72.54 mg/g), and its lowest in January 2020 (34.40 mg/g), September 2020 (45.41 mg/g), and October 2021 (33.57 mg/g) (*p* < 0.05).

Pearson correlation analysis revealed no significant associations among the 3D-anths (*p* > 0.05) or between 3D-anths content and the climatic variables, except for a moderate negative correlation between 3′-hydroxy-carajurone and mean relative humidity (*r* = −0.444; *p* < 0.05) (Supplementary Table S3).

The compound distribution in AC2 is compatible with a putative predominance of *O*-methylated products, including carajurin, and limited accumulation of the 3′-hydroxylated analogues. This compositional pattern does not demonstrate conversion of carajurone to carajurin, because enzyme expression, enzyme activity, metabolic flux, and precursor–product relationships were not measured (Figure 5).

In morphotype AC3 (Table 6), the level of 3′-hydroxy-carajurone content was the highest, ranging from 1.97 to 14.07 mg/g (*p* < 0.05). Carajurone (0.88–4.02 mg/g), 3′-hydroxy-carajurin (0.46–3.02 mg/g), and carajurin (0.28–2.40 mg/g) occurred at lower levels. AC3 was the only morphotype in which all four 3D-anths were detected in every sampled month. However, overlap among the content distributions made differentiation of AC3 from the other morphotypes difficult (*p* > 0.05), except for 3′-hydroxy-carajurone in 2020, whose content differed significantly among months and from that of the other compounds (*p* < 0.05). During 2020, 3′-hydroxy-carajurone ranged markedly (*p* < 0.01), with peaks in October (13.93 mg/g) and December (14.07 mg/g), whereas the other three compounds varied within narrower ranges (*p* > 0.05) (Figure 6C). Compound content declined substantially at the beginning of 2021 and subsequently remained within a narrow range. In 2021, differences in 3′-hydroxy-carajurone and carajurone content was not significant (*p* > 0.05).

Pearson correlation analysis revealed no significant associations between the compounds and the climatic variables over the two collection years (Supplementary Table S4). A strong positive correlation was observed between carajurone and 3′-hydroxy-carajurone (*r* = 0.990; *p* < 0.05), and another between 3′-hydroxy-carajurone and 3′-hydroxy-carajurin (*r* = 0.989; *p* < 0.05). These associations are consistent with related hydroxylation and *O*-methylation patterns but do not establish the direction of biosynthetic conversion.

As in AC3, 3′-hydroxy-carajurone was the predominant compound in AC4 (Table 7), ranging from 4.58 to 19.43 mg/g, followed by carajurone (1.15–4.59 mg/g); both were detected in every sampled month of 2020 and 2021. In contrast, 3′-hydroxy-carajurin (0.44–0.88 mg/g) and carajurin (0.36–1.78 mg/g) were detected only in four samples from 2020. 3′-Hydroxy-carajurone content was significantly higher than that of the other 3D-anths in both years (*p* < 0.05). This compound also showed marked temporal variation, which was more pronounced in 2020 than in 2021, resulting in different annual means (*p* < 0.05) (Figure 6D). The highest content levels occurred in June (19.43 mg/g) and October 2020 (14.58 mg/g), whereas the lowest occurred in January (4.70 mg/g) and December 2021 (4.58 mg/g). Carajurin and 3′-hydroxy-carajurin remained below 2 mg/g when detected and were absent throughout 2021.

A strong positive correlation was observed between carajurone and 3′-hydroxy-carajurone (*r* = 0.978; *p* < 0.05). By contrast, the low and sporadic content of carajurin and 3′-hydroxy-carajurin provides no evidence of consistently increased accumulation of these compounds. These associations are descriptive and do not demonstrate metabolic flux or biosynthetic conversion because enzyme regulation was not measured directly.

Pearson correlation analysis identified no significant associations between the 3D-anths and the climatic variables, except for a moderate negative correlation between 3′-hydroxy-carajurin and mean relative humidity (*r* = −0.493; *p* < 0.05) (Supplementary Table S5).

**Figure 7:**
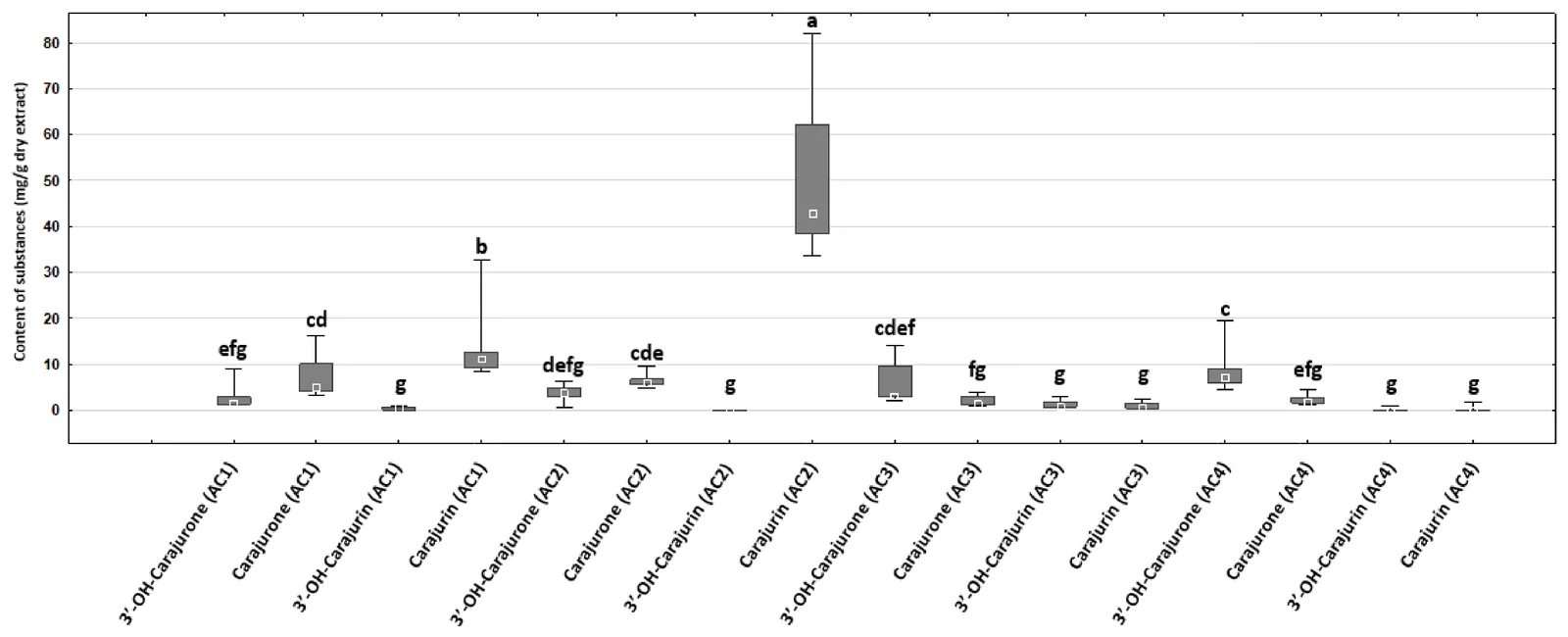
Box plots representing the percentage distributions of 3D-anths in the four *Fridericia chica* morphotypes collected during 2020 and 2021. Different letters indicate statistically significant differences.

In addition to the within-morphotype temporal comparisons, the four morphotypes were compared directly. Figure 7 presents box plots of the percentage distributions of the four 3D-anths in each *F. chica* morphotype.

Carajurin content differed significantly among morphotypes (*p* < 0.05), with AC2 showing the highest level of content, followed by AC1. Thus, AC1 and AC2 could be differentiated from AC3 and AC4 by their carajurin content. Comparison between collection months showed that carajurin was the major compound in AC1 and AC2 (Figure 6A,B), whereas 3′-hydroxy-carajurone predominated in AC3 and AC4 (Figure 6C,D) (*p* < 0.05). Variations in the major compounds were generally accompanied by proportional variations in the minor compounds. Across the two collection years, AC1 and AC2 were differentiated by carajurin content, whereas AC4 was distinguished by its 3′-hydroxy-carajurone content (*p* < 0.05).

#### 2.2.3. Chemometric Differentiation of *F. chica* Morphotypes Based on 3D-Anths Profiles

**Figure 8:**
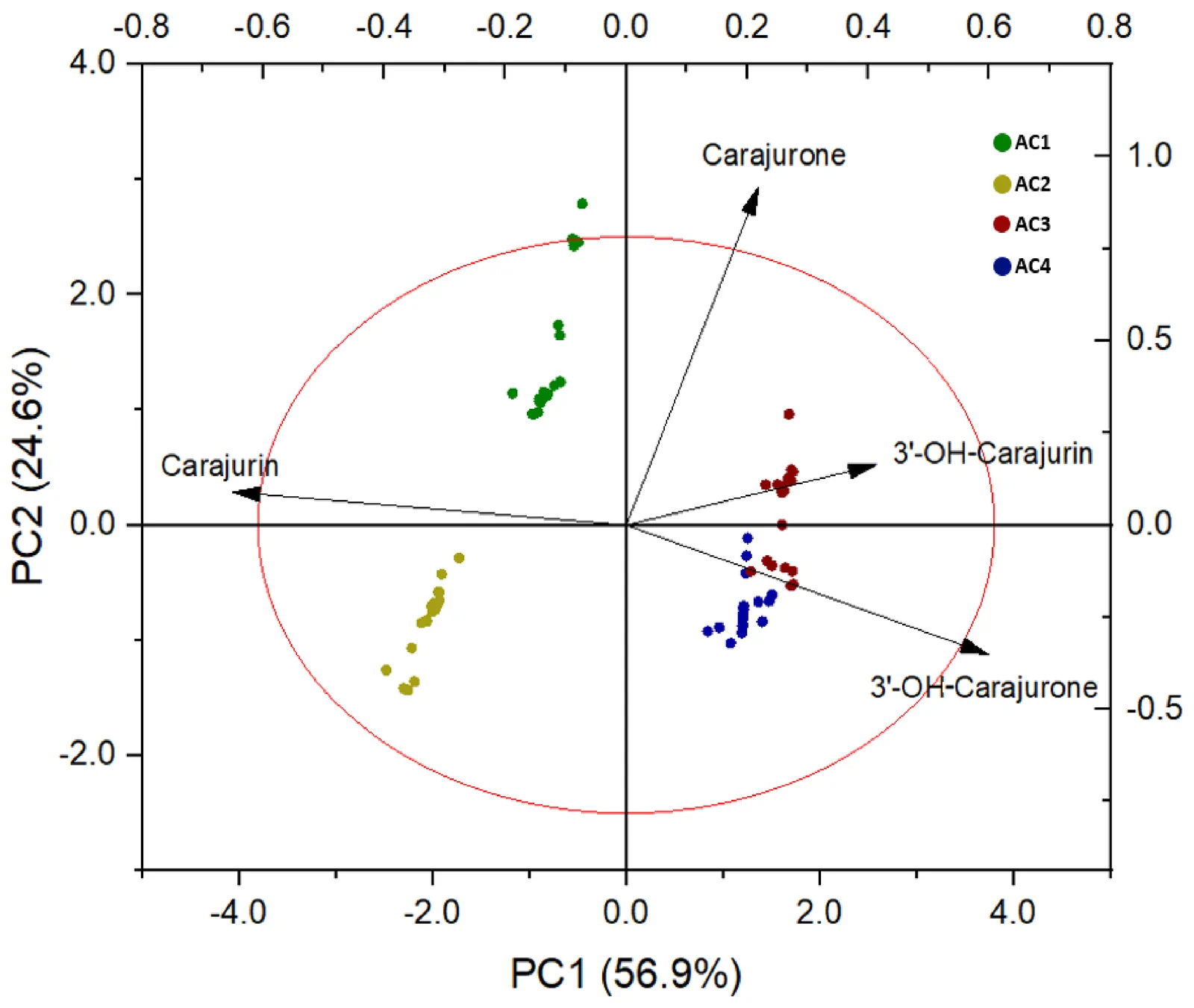
Principal component analysis (PCA) of extracts from the four morphotypes of *Fridericia chica*.

The principal component analysis (PCA) model explained 81.5% of the total variance (Figure 8). The first principal component (PC1; 56.9% of the variance) was characterized by a negative loading for carajurin (−0.65) and a positive loading for 3′-hydroxy-carajurone (0.60). These loadings placed AC3 and AC4 mainly at positive PC1 scores and AC1 and AC2 at negative scores. PC2 was influenced by a positive loading for carajurone (0.92) and a negative loading for 3′-hydroxy-carajurone (−0.35), placing AC1 mainly at positive PC2 scores and AC2 and AC4 at negative scores. For AC3, most 2021 samples had positive PC2 scores, whereas most 2020 samples had negative scores; the September sample was located near the PC2 origin. The score plot suggested at least three groups: AC1 and AC2 formed well-defined groups, whereas AC3 and AC4 were closer and partially overlapped.

**Figure 9:**
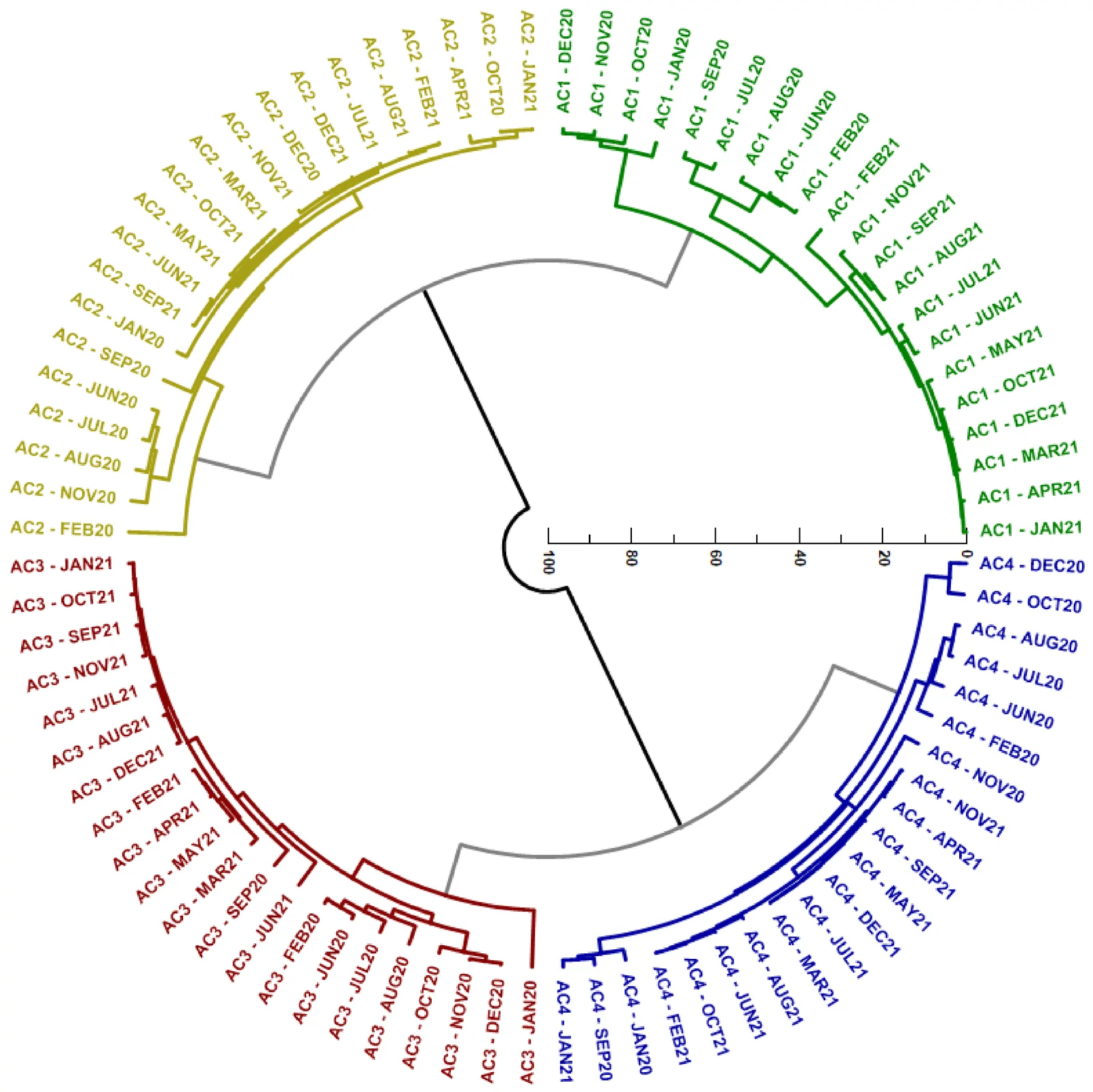
Hierarchical cluster analysis (HCA) of extracts from the four morphotypes of *Fridericia chica*.

The pattern described above was also examined by hierarchical cluster analysis (HCA). HCA yielded four distinct groups whose membership was consistent with the predefined classification of the four *F. chica* morphotypes in the present dataset (Figure 9). Each cluster contained the monthly samples assigned to one morphotype, and colors were used to facilitate visualization. A greater Euclidean distance separated AC1 and AC2 from AC3 and AC4. Because no external classification or resampling validation was performed, this result is interpreted as exploratory evidence of chemical differentiation.

Four main clusters were identified: cluster 1 (green) corresponded to AC1, cluster 2 (yellow) to AC2, cluster 3 (red) to AC3, and cluster 4 (blue) to AC4. Each cluster was further subdivided into smaller subclusters.

In this dataset, the four clusters were consistent with the four evaluated morphotypes, indicating that their 3D-anths profiles provided exploratory chemical differentiation under the conditions studied.

**Figure 10:**
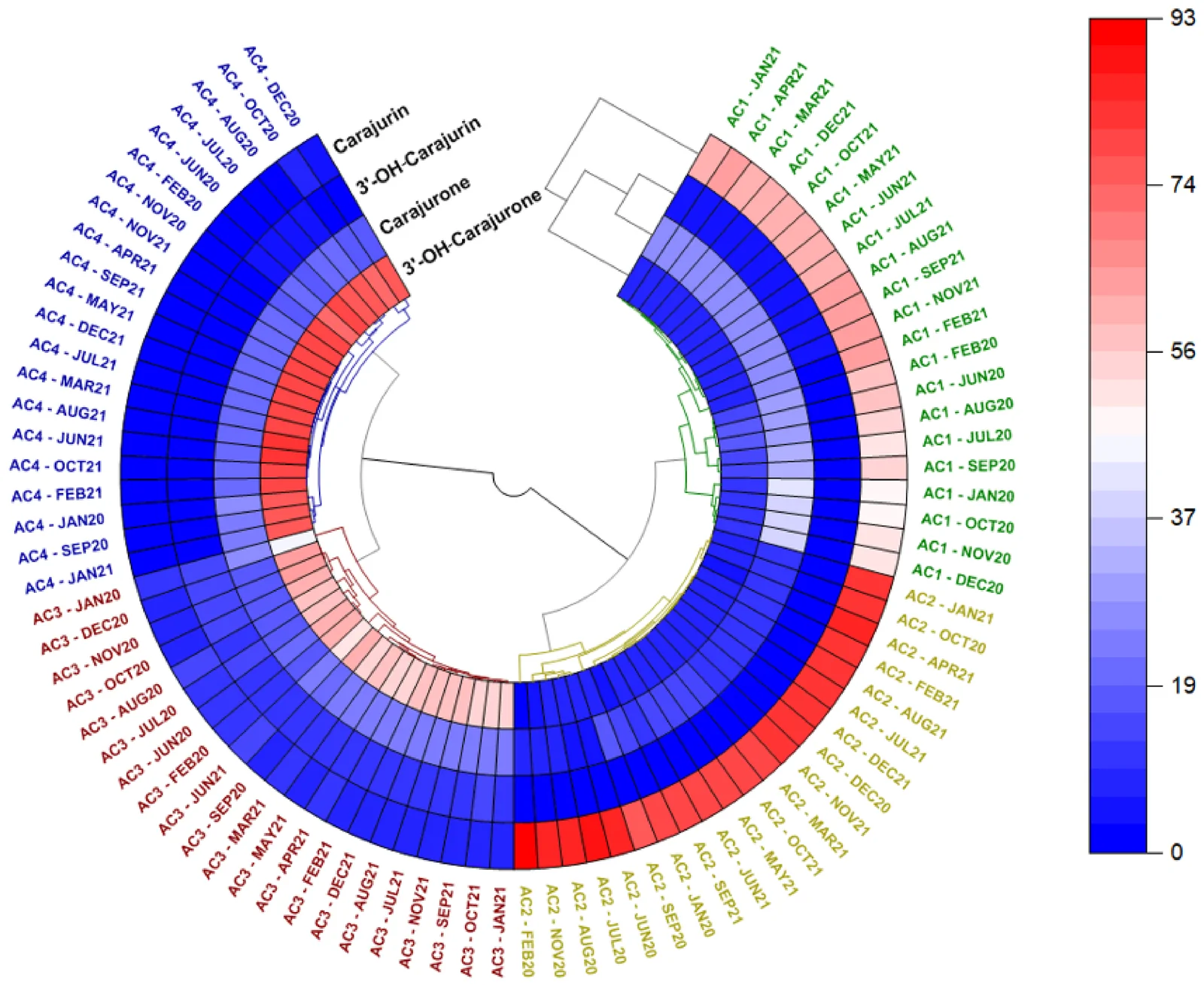
Circular heat map grouped with a dendrogram of the extracts from the four morphotypes of *Fridericia chica.* Colors indicate the quantities of the 3D-anths studied, according to the legend.

The circular heat map (Figure 10), which includes the HCA dendrogram, displays the content levels of the four 3D-anths in each of the 84 samples collected during 2020–2021. In AC1 and AC2, corresponding to HCA groups 1 and 2, carajurin was predominant, especially in group 2. In AC3 and AC4, corresponding to groups 3 and 4, compound 3′-hydroxy-carajurone was the major 3D-anths, with the greatest predominance in group 4.

Although PCA and HCA have different objectives, they are complementary tools for exploratory data analysis and identification of relationships and patterns [48]. PCA was used primarily to characterize the major sources of variability in the 3D-anths composition of *F. chica* extracts, whereas HCA grouped samples according to similarity. PCA suggested at least three partially distinct groups, while HCA resolved four clusters corresponding to the four morphotypes. These results emphasize the need to establish appropriate acceptance ranges for marker compounds while accounting for seasonal and ecological variation.

The PCA and HCA results therefore emphasize the importance of carefully defining acceptance ranges for marker-compound content in different *F. chica* morphotypes. Relating these ranges to ecological factors may provide additional criteria for quality control. Molecular networking platforms such as Global Natural Products Social Molecular Networking (GNPS) may also help identify additional constituents that contribute to differentiation among chemical profiles [49].

Together with the previously developed carajurin-based method for 3D-anths quantification [11], these findings support future standardization of a plant-based active pharmaceutical ingredient (API) from crude *F. chica* extracts. Although AC3 consistently contained all four 3D-anths in every collection month, the mean levels of content of these compounds did not clearly distinguish it from the other morphotypes. Additional chemical markers may therefore be required. The results indicate the possible occurrence of distinct chemical phenotypes and provide additional criteria for quality control of the species.

These findings are consistent with previous studies showing substantial chemical variability in medicinal plants, driven by multiple environmental and intrinsic factors, and its relevance to phytomedicine development and production [50]. Specialized-metabolite production is known to be influenced by climatic conditions [50,51,52]. The complexity of this relationship increases because each plant species has its own metabolic characteristics [50,51]. Consequently, environmental disturbances may trigger species-specific phenotypic chemical plasticity.

In the present study, temporal variation in 3D-anths content was not consistently explained by the measured climatic variables. Moderate negative correlations with relative humidity were observed for carajurin in AC1, 3′-hydroxy-carajurone in AC2, and 3′-hydroxy-carajurin in AC4, whereas most associations with temperature, dew point, pressure, wind speed, radiation, and precipitation were not significant. These morphotype-specific patterns may reflect differences in physiological regulation or unmeasured environmental and developmental factors, but the present data do not permit attribution to genetic or metabolic mechanisms because these variables were not measured directly.

The observed temporal variation did not show strong associations with the measured climatic variables, consistent with some previous studies [53,54,55]. In *Vitis vinifera* L. (Vitaceae), both cultivar and climatic conditions have been shown to alter anthocyanin composition substantially [56]. Anthocyanin production is also strongly regulated at the enzymatic level. However, in blueberry, anthocyanin composition was closely associated with differential expression of biosynthetic enzymes [57].

Environmental factors may also influence the enzymatic regulation of anthocyanidin production [54]. Although the present results indicate that anthocyanidin production in *F. chica* was not explained exclusively by the measured environmental variables, temperature, light, and water availability may affect enzymes involved in flavonoid biosynthesis [54,58]. Further ecological, biochemical, and genetic studies are therefore needed to clarify regulation of anthocyanidin production in the evaluated morphotypes.

## 3. Materials and Methods

### 3.1. Plant Material Collection and Identification

Four *Fridericia chica* morphotypes were cultivated and sampled monthly between January 2020 and December 2021 (approximately 1 kg of plant material from each morphotype per month), consistently between 9:00 and 10:00 a.m. during the first week of each month, at the Farmanguinhos/Fiocruz Atlantic Forest Campus, Rio de Janeiro, RJ, Brazil (22.9406° S, 43.4046° W). Collections were not performed in March, April, or May 2020 because of the COVID-19 pandemic. The morphotypes, designated AC1, AC2, AC3, and AC4, were identified and classified by Marcelo Galvão Neto (Farmanguinhos/Fiocruz). Voucher specimens were deposited in the Botanical Collection of Medicinal Plants (CBPM) under accession numbers CBPM 665, 666, 667, and 668, respectively. Access to Brazilian genetic heritage was registered with the Genetic Heritage Management Council under code A670412.

Climatological data corresponding to the monthly collections, including solar radiation, mean temperature, relative humidity, and accumulated precipitation, were obtained from the National Institute of Meteorology (INMET) automatic weather station located at the Atlantic Forest Campus (station A636).

*F. chica* leaves were dried in a forced-air oven at 45 °C until constant mass (approximately 4 days) and ground in a knife mill. The powdered material was standardized by passage through a 32-mesh sieve (<500 µm) and stored in sealed, light-resistant plastic bags protected from moisture.

### 3.2. Design of Experiments (DoE)

#### 3.2.1. Experimental Design

To optimize extraction of 3D-anths from *F. chica* leaves, a factorial experimental design was conducted using morphotype AC2, selected because of its high carajurin content. The effects of ethanol concentration in the extraction solution and ultrasonic-bath extraction time (model Q335D; Quimis, Diadema, Brazil) on extract yield, 3D-anths content, and antileishmanial responses were evaluated.

Nine extraction conditions were evaluated using two independent variables: extraction time (*X*1; 15, 30, and 45 min) and ethanol concentration in ultrapure water (*X*2; 70%, 80%, and 100%; Merck, Darmstadt, Germany) (Table 8 and Table 9). The −1, 0, and +1 entries in the tables identify the low, intermediate, and high experimental levels; because ethanol levels were unequally spaced in physical units, the actual ethanol concentrations (70, 80, and 100%) were used in polynomial regression. The dependent variables were extract yield; 3D-anths content, expressed as carajurin equivalents and quantified using a validated HPLC-DAD method [11]; IC_50_; CC_50_; and SI (Section 3.4).

For each condition, three independently prepared extraction replicates were analyzed and summarized as mean ± standard deviation. The regression used the mean response for each of the nine conditions; consequently, total DF was 8. The three DF values reported in Table 2 represent residual error after fitting the six model coefficients and should not be interpreted as pure experimental error. Analysis of variance (ANOVA) and second-order polynomial regression, including linear, quadratic, and interaction terms, were performed in Statistica version 13 (StatSoft Inc., Tulsa, OK, USA).

A polynomial model was fitted to describe the relationship between the independent variables (*X*1 and *X*2) and each response (*Y*), as follows:

$$Y=b0+b1X1+b2X2+b3{X1}^{2}+b4{X2}^{2}+b5X1X2$$

In this equation, *Y* is the response (extract yield, 3D-anths content, IC_50_, CC_50_, or SI), *X*1 is extraction time, *X*2 is ethanol concentration, *b*0 is the intercept, *b*1 and *b*2 are the linear coefficients, *b*3 and *b*4 are the quadratic coefficients, and *b*5 is the interaction coefficient.

The coefficient of determination (*R*^2^) was used to assess model fit. Regression models and coefficients were considered statistically significant at *p* < 0.05.

Pareto charts and response surface methodology (RSM) were used to visualize factor effects. No automated desirability function was applied. The final condition was chosen by supervised multi-criteria assessment: recovery of carajurin and total 3D-anths was treated as the primary standardization criterion, while extract yield, extraction time, IC_50_, CC_50_, and SI were considered jointly. Biological differences were not treated as decisive unless statistically supported [59].

#### 3.2.2. Extraction of Plant Material for the Experimental Design

For each assay, 1.0 g of dried and ground AC2 plant material, selected based on preliminary tests, was transferred to a screw-capped tube. The corresponding extraction solution (10 mL) was added at a plant-to-solvent ratio of 1:10 (*w*/*v*). The sealed tubes were extracted in a Q335D ultrasonic bath (Quimis, Diadema, Brazil; nominal ultrasonic frequency 40 kHz; ultrasonic power 135 W RMS; tank capacity 2.8 L) for the amount of time specified in the design. Extractions were performed at room temperature without active temperature control; bath temperature was not recorded, which limits mechanistic interpretation of possible temperature-dependent effects. The extracts were filtered through qualitative filter paper, and the solvent was evaporated under airflow in a fume hood. Dry extracts were weighed and stored at 0–5 °C until analysis. Extract yield was calculated relative to initial plant mass. 3D-anths content was calculated relative to dry-extract mass and expressed as carajurin equivalent [6]. Each condition comprised three independently prepared extraction replicates, yielding 27 extracts.

#### 3.2.3. Chromatographic Analysis of Extracts Produced in the Experimental Design

Each dry crude extract (10 mg) was dissolved in 1 mL of the corresponding extraction solution (70%, 80%, or 100% ethanol in ultrapure water), sonicated for 15 min, filtered through a 0.45 µm membrane (Sigma-Aldrich, St. Louis, MO, USA), and analyzed by HPLC-DAD. When necessary, the 10 mg/mL solution was diluted so that peak areas remained within the calibration range obtained with isolated carajurin (98% HPLC purity) [6]. The previously validated method [11] established calibration, linearity, precision, accuracy, detection and quantification limits, and system-suitability criteria. In the present study, retention times and UV-visible spectra were checked against the characterized carajurin standard and the validated chromatographic profile. Because carajurin was the only available reference standard, the other 3D-anths were reported as carajurin equivalents; possible differences in detector response among compounds are therefore acknowledged as a limitation.

### 3.3. Seasonal and Statistical Analysis of Fridericia chica Extracts from Monthly Collections

#### 3.3.1. Initial Procedures

After determining the optimal conditions for 3D-anths extraction, the plant material was analyzed for seasonal variability. Samples were collected monthly during 2020 and 2021, consistently between 9:00 and 10:00 a.m. during the first week of each month. The selected specimens were cultivated under comparable conditions and showed no visible leaf damage caused by herbivory. Further details on plant collection and drying are provided in Section 3.1. Chromatographic analyses were performed as described for the DoE extracts [11].

#### 3.3.2. Data Processing and Analysis

Chemometric analyses were conducted to investigate relationships, correlations, congruence, and variation among the extracts and their respective 3D-anths profiles in the four *F. chica* morphotypes. The analyses evaluated: (1) differences in chemical composition among morphotypes; (2) the influence of climatic variables, including temperature (°C), relative humidity (%), dew point (°C), atmospheric pressure (hPa), wind speed (m/s), solar radiation (kJ/m^2^), and accumulated precipitation (mm); and (3) seasonal variation, particularly between dry and rainy periods. Climatological data were obtained from the National Institute of Meteorology automatic weather station nearest the cultivation site (A636; WMO 83743).

#### 3.3.3. Descriptive Analysis of Data and Variation

For the seasonal study, each monthly morphotype sample consisted of a bulk collection of leaves from the cultivated specimen described in Section 3.1. The reported triplicates were replicate HPLC determinations of the corresponding extract and therefore represent technical analytical replication, not independent biological replication. Results are expressed as mean ± standard deviation. Data normality was assessed using the Shapiro–Wilk test [60,61]. For datasets satisfying the normality assumption, ANOVA followed, when appropriate, by Tukey’s test was used at a 5% significance level. In Table 4, Table 5, Table 6 and Table 7, superscript letters compare monthly values within each morphotype table; values without a shared letter differ significantly (*p* < 0.05). Analyses were performed in Statistica version 12 (StatSoft Inc., Tulsa, OK, USA). The absence of independent plants within each morphotype limits inference beyond the sampled cultivated specimens.

#### 3.3.4. Correlation Analysis

Pearson correlation analysis was used to explore associations between climatic variables and 3D-anths content [61]. Absolute coefficients were interpreted according to [62]: 0.00–0.19, very weak; 0.20–0.39, weak; 0.40–0.69, moderate; 0.70–0.89, strong; and 0.90–1.00, very strong. Significance was assessed using Student’s *t*-test. Because numerous correlated tests were performed and no multiplicity adjustment was applied, climatic and within-DoE correlation analyses were considered exploratory; isolated *p*-values were not interpreted as confirmatory evidence.

#### 3.3.5. Multivariate Analysis

Principal component analysis (PCA) and hierarchical cluster analysis (HCA) were conducted using Statistica version 12 (StatSoft Inc., Tulsa, OK, USA) and OriginPro 2023 (OriginLab Corp., Northampton, MA, USA).

Correlation matrices were constructed from the 3D-anths composition of *F. chica* extracts. HPLC-DAD percentage-area values were normalized to 100%, mean-centered, and scaled to unit variance before PCA and HCA [6].

For HCA, Euclidean distances and the unweighted pair-group method with arithmetic mean (UPGMA) were applied to the same autoscaled matrix used for PCA [6]. A circular heat map combined with the dendrogram was used as an exploratory visualization. No external classification set or resampling validation was used.

### 3.4. In Vitro Antileishmanial Activity Assays

#### 3.4.1. Antipromastigote Assay

Promastigotes of *L. amazonensis* (10^6^ parasites/mL) from 3–5-day-old cultures were seeded in 96-well plates and incubated for 72 h with *F. chica* crude extracts (3.9–500 μg/mL), 3D-anths-enriched fractions (1.95–500 μg/mL), or carajurin (0.78–100 μg/mL). Amphotericin B (0.01–2.5 μg/mL) was used as the reference drug, and the final volume was 100 μL per well. Wells without parasites served as blanks, and wells containing parasites without treatment served as controls. Parasite viability was assessed by counting motile promastigotes in a Neubauer chamber under optical microscopy using a 40× objective. Data were normalized as follows: survival (%) = sample count/control count × 100. The results were used to calculate the concentration that inhibited parasite growth by 50% (IC_50_) [29]. All assays were performed in at least three independent biological experiments (n ≥ 3), each with technical triplicates. IC_50_ values were estimated by nonlinear regression using a concentration–response inhibition model in GraphPad Prism version 8.0.1 (GraphPad Software, San Diego, CA, USA). Under these conditions, amphotericin B yielded an IC_50_ of 0.12 ± 0.02 µg/mL.

#### 3.4.2. Cytotoxicity Assay

Peritoneal macrophages from female BALB/c mice aged 4–6 weeks, obtained from the Institute of Science and Technology in Biomodels of the Oswaldo Cruz Foundation (CEUA-IOC; protocol L53/2016-A3), were seeded in 96-well plates (5 × 10^5^ cells/mL) and exposed to *F. chica* crude extracts (7.81–1000 μg/mL), 3D-anths-enriched fractions (7.81–1000 µg/mL), carajurin (3.9–500 μg/mL), or amphotericin B (0.19–25 μg/mL) for 24 h in a final volume of 100 μL per well at 37 °C and 5% CO_2_. Wells without cells served as blanks, and wells containing cells with 1% DMSO served as controls. Cell viability was assessed by adding 10 μL of MTT solution (5 mg/mL); after 2 h, the medium was removed and 100 μL DMSO was added to dissolve the formazan crystals. Absorbance was normalized to the untreated control. CC_50_ values were estimated by nonlinear concentration–response regression in GraphPad Prism version 8.0.1. All assays comprised at least three independent biological experiments (n ≥ 3), each with technical triplicates. Amphotericin B yielded a CC_50_ of 12.54 ± 1.15 µg/mL and an SI of 104.5. The 72 h parasite exposure and 24 h macrophage exposure reflect the different kinetics of the models: the longer interval permits assessment across multiple promastigote replication cycles, whereas the shorter interval evaluates acute effects in primary, non-dividing host cells while reducing culture-related metabolic artifacts [29,63]. Because cytotoxicity is time-dependent, SI values based on different exposure periods should be interpreted as screening indices rather than direct time-matched therapeutic windows. SI was calculated as CC_50_/IC_50_.

## 4. Conclusions

Within the evaluated experimental domain, 30 min and 100% ethanol (assay F) provided the highest total 3D-anths content (12.58%) and carajurin recovery (10.37%) while retaining antileishmanial activity (IC_50_ = 7.22 µg/mL; SI = 7.5). This condition was selected by a supervised multi-criteria assessment that prioritized active-marker recovery and process standardization; no formal desirability function was applied.

Application of this condition to monthly samples revealed substantial temporal variation in 3D-anths content. Carajurin predominated in AC1 and AC2, particularly in AC2, whereas 3′-hydroxy-carajurone predominated in AC3 and AC4. Most associations with the measured climatic variables were not statistically significant, although moderate negative correlations with relative humidity occurred for selected metabolites in AC1, AC2, and AC4. Thus, the observed variation was not consistently explained by the climatic variables evaluated, and no inference regarding genetic or metabolic causation can be made from the present data.

Exploratory PCA and HCA indicated chemical differentiation among the four morphotypes based on their 3D-anths profiles. Although quantitative contents varied over time, the characteristic chromatographic profile of each sampled morphotype remained comparatively consistent across collection months. These results apply to the cultivated specimens studied and require validation with independent biological samples.

Together with previous findings, these results provide a basis for future studies on the development and standardization of a plant-based active pharmaceutical ingredient from *F. chica*. Such development will require independent biological replication, time-matched safety assessment, confirmation in clinically relevant parasite stages and in vivo models, and further chemical, pharmacological, and process validation; therapeutic efficacy was not demonstrated in the present study.

## Figures and Tables

**Table 1:** Experimental design (DoE) for optimization of the extraction process from *Fridericia chica* leaves (AC2 morphotype).

Assay	Independent Variables		Dependent Variables (Value ± Standard Deviation)						
	*X*1	*X*2	Yield (%)	3′-Hydroxy- Carajurone (%)	Carajurone (%)	Carajurin (%)	IC_50_/72 h (µg/mL)	CC_50_/24 h (µg/mL)	SI
A	15	70	30.80 ± 0.72	0.11 ± 0.00	0.17 ± 0.02	0.83 ± 0.01	111.90 ± 1.12	655.10 ± 1.24	5.9
B	15	80	28.28 ± 0.70	0.13 ± 0.00	0.18 ± 0.00	1.03 ± 0.01	104.80 ± 1.17	569.20 ± 1.22	5.4
C	15	100	15.59 ± 0.29	0.66 ± 0.01	1.03 ± 0.01	7.47 ± 0.07	6.09 ± 1.10	112.60 ± 1.18	18.5
D	30	70	28.06 ± 0.49	0.43 ± 0.01	0.56 ± 0.01	4.13 ± 0.04	44.01 ± 1.19	113.40 ± 1.26	2.6
E	30	80	26.27 ± 0.30	0.49 ± 0.01	0.71 ± 0.00	4.96 ± 0.07	40.60 ± 1.35	173.70 ± 1.17	4.3
F	30	100	16.23 ± 0.63	0.85 ± 0.00	1.36 ± 0.00	10.37 ± 0.11	7.22 ± 1.09	53.76 ± 1.17	7.5
G	45	70	32.19 ± 1.04	0.23 ± 0.00	0.31 ± 0.00	2.23 ± 0.03	75.87 ± 1.21	291.20 ± 1.28	3.8
H	45	80	22.22 ± 0.77	0.30 ± 0.02	0.43 ± 0.00	3.05 ± 0.03	72.49 ± 1.37	403.00 ± 1.14	5.6
I	45	100	17.58 ± 0.46	0.83 ± 0.00	1.35 ± 0.02	9.44 ± 0.07	9.87 ± 1.18	58.27 ± 1.15	5.9
AmB *	—	—	—	—	—	—	0.12 ± 0.02	12.54 ± 1.15	104.5

Legend: *X*1 = extraction time (min); *X*2 = ethanol concentration in ultrapure water (%); IC_50_ = concentration inhibiting 50% of parasites after 72 h; CC_50_ = concentration reducing macrophage viability by 50% after 24 h; SI = CC_50_/IC_50_. Values are mean ± standard deviation from at least three independent biological experiments conducted in technical triplicate. * AmB = Amphotericin B (positive reference control for parasite and cell-viability assays).

**Table 2:** Analysis of variance (ANOVA) for the evaluated dependent variables.

**Factors**	**Extract Yield (%)**				
	**SS**	**DF**	**MS**	***F*** **-Value**	***p*** **-Value**
(*X*1) Extraction time (min) (L)	0.871	1	0.871	0.102	0.771
(*X*1) Extraction time (min) (Q)	1.705	1	1.705	0.199	0.686
(*X*2) EtOH concentration (%) (L)	289.120	1	289.120	33.685	0.010 *
(*X*2) EtOH concentration (%) (Q)	0.034	1	0.034	0.004	0.954
Linear interactions (*X*1L *by* *X*2L)	1.300	1	1.300	0.151	0.723
Residual error	25.750	3	8.583		
Total	328.591	8			
**Factors**	**3′-Hydroxy-Carajurone (%)**				
	**SS**	**DF**	**MS**	***F*** **-Value**	***p*** **-Value**
(*X*1) Extraction time (min) (L)	0.037	1	0.037	9.445	0.054
(*X*1) Extraction time (min) (Q)	0.089	1	0.089	22.777	0.017 *
(*X*2) EtOH concentration (%) (L)	0.415	1	0.415	106.496	0.002 *
(*X*2) EtOH concentration (%) (Q)	0.030	1	0.030	7.691	0.069
Linear interactions (*X*1L *by X*2L)	0.001	1	0.001	0.157	0.718
Residual error	0.012	3	0.004		
Total	0.642	8			
**Factors**	**Carajurone (%)**				
	**SS**	**DF**	**MS**	***F*** **-Value**	***p*** **-Value**
(*X*1) Extraction time (min) (L)	0.092	1	0.092	14.630	0.032 *
(*X*1) Extraction time (min) (Q)	0.181	1	0.181	28.856	0.013 *
(*X*2) EtOH concentration (%) (L)	1.205	1	1.205	191.697	0.001 *
(*X*2) EtOH concentration (%) (Q)	0.083	1	0.083	13.202	0.036 *
Linear interactions (*X*1L *by X*2L)	0.008	1	0.008	1.203	0.353
Residual error	0.019	3	0.006		
Total	1.754	8			
**Factors**	**Carajurin (%)**				
	**SS**	**DF**	**MS**	***F*** **-Value**	***p*** **-Value**
(*X*1) Extraction time (min) (L)	5.220	1	5.220	27.712	0.013 *
(*X*1) Extraction time (min) (Q)	11.530	1	11.530	61.213	0.004 *
(*X*2) EtOH concentration (%) (L)	68.533	1	68.533	363.855	0.0003 *
(*X*2) EtOH concentration (%) (Q)	4.921	1	4.921	26.129	0.014 *
Linear interactions (*X*1L *by X*2L)	0.126	1	0.126	0.667	0.474
Residual error	0.565	3	0.188		
Total	100.690	8			
**Factors**	**IC_50_/72 h (µg/mL)**				
	**SS**	**DF**	**MS**	***F*** **-Value**	***p*** **-Value**
(*X*1) Extraction time (min) (L)	542.84	1	542.845	1.532	0.304
(*X*1) Extraction time (min) (Q)	2163.96	1	2163.965	6.107	0.090
(*X*2) EtOH concentration (%) (L)	7252.05	1	7252.049	20.467	0.020 *
(*X*2) EtOH concentration (%) (Q)	663.44	1	663.435	1.872	0.265
Linear interactions (*X*1L *by X*2L)	454.33	1	454.328	1.282	0.340
Residual error	1062.97	3	354.324		
Total	13,444.23	8			
**Factors**	**CC_50_/24 h (µg/mL)**				
	**SS**	**DF**	**MS**	***F*** **-Value**	***p*** **-Value**
(*X*1) Extraction time (min) (L)	47,079.6	1	47,079.6	3.153	0.174
(*X*1) Extraction time (min) (Q)	110,082.1	1	110,082.1	7.372	0.073
(*X*2) EtOH concentration (%) (L)	116,223.7	1	116,223.7	7.783	0.068
(*X*2) EtOH concentration (%) (Q)	28,478.9	1	28,478.9	1.907	0.261
Linear interactions (*X*1L *by X*2L)	21,701.3	1	21,701.3	1.453	0.314
Residual error	44,800.3	3	14,933.4		
Total	406,122.4	8			
**Factors**	**SI**				
	**SS**	**DF**	**MS**	***F*** **-Value**	***p*** **-Value**
(*X*1) Extraction time (min) (L)	44.348	1	44.348	8.803	0.059
(*X*1) Extraction time (min) (Q)	14.761	1	14.761	2.930	0.186
(*X*2) EtOH concentration (%) (L)	64.027	1	64.027	12.709	0.038 *
(*X*2) EtOH concentration (%) (Q)	2.675	1	2.675	0.531	0.519
Linear interactions (*X*1L *by X*2L)	35.751	1	35.751	7.097	0.077
Residual error	15.114	3	5.038		
Total	174.969	8			

Legend: Q = quadratic; L = linear; SS = sum of squares; DF = degrees of freedom; MS = mean square; SI = selectivity index; EtOH = ethanol. * Statistically significant values (*p* < 0.05).

**Table 3:** Models generated by multiple regression for the dependent variables as functions of *X*1 (extraction time) and *X*2 (ethanol concentration), with their coefficients of determination.

Equation	Generated Model	*R* ^2^
(1)	$Extract\;yield\;(\%)=77.564-0.483\times X_{1}+0.00410\times {X_{1}}^{2}-0.650\times X_{2}+0.000661\times {X_{2}}^{2}+0.00249\times X_{1}\times X_{2}$	0.922
(2)	$3^{\prime }-hydroxy-Carajurone\;\left(\%\right)=2.670+0.0569\times X_{1}-0.000936\times {X_{1}}^{2}-0.0901\times X_{2}+0.000623\times {X_{2}}^{2}+0.0000540\times X_{1}\times X_{2}$	0.982
(3)	$Carajurone\;\left(\%\right)=4.745+0.0725\times X_{1}-0.00134\times {X_{1}}^{2}-0.152\times X_{2}+0.00104\times {X_{2}}^{2}+0.000190\times X_{1}\times X_{2}$	0.989
(4)	$Carajurin\;\left(\%\right)=34.586+0.637\times X_{1}-0.0107\times {X_{1}}^{2}-1.158\times X_{2}+0.00799\times {X_{2}}^{2}+0.000773\times X_{1}\times X_{2}$	0.994
(5)	${IC}_{50}\;\left(µg|/|mL\right)=-162.209-13.365\times X_{1}+0.146\times {X_{1}}^{2}+12.052\times X_{2}-0.0927\times {X_{2}}^{2}+0.0465\times X_{1}\times X_{2}$	0.921
(6)	${CC}_{50}\;\left(µg|/|mL\right)=-1469.930-95.845\times X_{1}+1.0427\times {X_{1}}^{2}+84.369\times X_{2}-0.608\times {X_{2}}^{2}+0.321\times X_{1}\times X_{2}$	0.890
(7)	$SI=11.348+0.202\times X_{1}+0.0121\times {X_{1}}^{2}-0.392\times X_{2}+0.00589\times {X_{2}}^{2}-0.0130\times X_{1}\times X_{2}$	0.914

Legend: *R*^2^ = coefficient of determination; *X*1 = extraction time; *X*2 = ethanol concentration; SI = selectivity index.

**Table 4:** Quantification of 3D-anths in crude ethanolic extracts of morphotype AC1 collected monthly during 2020 and 2021.

Collection Year	Collection Month	Substance Content (mg/g Dry Extract) ± Standard Deviation (SD)			
		3′-Hydroxy- Carajurone	Carajurone	3′-Hydroxy- Carajurin	Carajurin
2020	JAN	2.35 ± 0.02 ^H^	10.17 ± 0.02 ^o^	N.D.	11.86 ± 0.11 ^kL^
	FEB	8.84 ± 0.08 ^st^	16.20 ± 0.11 ^f^	N.D.	32.83 ± 0.07 ^a^
	JUN	8.44 ± 0.05 ^v^	15.00 ± 0.07 ^g^	N.D.	30.27 ± 0.06 ^b^
	JUL	5.38 ± 0.03 ^y^	11.85 ± 0.07 ^L^	N.D.	18.95 ± 0.11 ^d^
	AUG	6.59 ± 0.03 ^x^	11.75 ± 0.03 ^L^	N.D.	22.95 ± 0.10 ^c^
	SEP	4.36 ± 0.01 ^C^	10.61 ± 0.02 ^n^	N.D.	16.84 ± 0.09 ^e^
	OCT	2.94 ± 0.01 ^G^	9.78 ± 0.07 ^p^	N.D.	12.24 ± 0.04 ^i^
	NOV	2.24 ± 0.01 ^HI^	8.66 ± 0.06 ^tu^	N.D.	11.18 ± 0.07 ^m^
	DEC	2.05 ± 0.00 ^I^	7.25 ± 0.05 ^w^	N.D.	9.37 ± 0.02 ^q^
2021	JAN	1.55 ± 0.01 ^J^	5.14 ± 0.04 ^z^	0.69 ± 0.00 ^OPQR^	12.04 ± 0.04 ^jk^
	FEB	1.22 ± 0.01 ^LM^	4.07 ± 0.02 ^DE^	N.D.	9.38 ± 0.01 ^q^
	MAR	1.19 ± 0.00 ^MN^	3.93 ± 0.03 ^E^	0.59 ± 0.00 ^QR^	9.17 ± 0.03 ^r^
	APR	1.20 ± 0.05 ^MN^	3.98 ± 0.02 ^E^	0.60 ± 0.00 ^QR^	9.47 ± 0.01 ^q^
	MAY	1.23 ± 0.00 ^LM^	3.90 ± 0.01 ^E^	0.61 ± 0.00 ^PQR^	8.87 ± 0.02 ^s^
	JUN	1.33 ± 0.01 ^KLM^	4.25 ± 0.02 ^CD^	0.70 ± 0.00 ^OPQR^	9.29 ± 0.05 ^qr^
	JUL	1.32 ± 0.01 ^KLM^	4.32 ± 0.02 ^C^	0.82 ± 0.00 ^O^	9.35 ± 0.36 ^qr^
	AUG	1.40 ± 0.00 ^JKL^	4.93 ± 0.02 ^A^	0.77 ± 0.00 ^OPQ^	12.55 ± 0.09 ^h^
	SEP	1.32 ± 0.00 ^KLM^	4.73 ± 0.02 ^B^	0.73 ± 0.00 ^OPQR^	12.05 ± 0.06 ^ij^
	OCT	1.42 ± 0.01 ^JK^	4.78 ± 0.03 ^AB^	0.80 ± 0.00 ^OP^	11.10 ± 0.07 ^m^
	NOV	1.02 ± 0.00 ^N^	3.41 ± 0.03 ^F^	0.56 ± 0.00 ^R^	8.52 ± 0.03 ^uv^
	DEC	1.16 ± 0.00 ^MN^	4.00 ± 0.01 ^E^	0.58 ± 0.00 ^QR^	9.17 ± 0.03 ^r^

Within each morphotype table, values without a shared superscript letter differ significantly according to Tukey’s test (*p* < 0.05); N.D. = not detected. Letters refer to comparisons across collection months for the reported metabolite values.

**Table 5:** Quantification of 3D-anths in crude ethanolic extracts of morphotype AC2 collected monthly during 2020 and 2021.

Collection Year	Collection Month	Substance Content (mg/g Dry Extract) ± Standard Deviation (SD)			
		3′-Hydroxy- Carajurone	Carajurone	3′-Hydroxy- Carajurin	Carajurin
2020	JAN	2.63 ± 0.02 ^L^	6.18 ± 0.01 ^yz^	N.D.	34.40 ± 0.15 ^s^
	FEB	6.40 ± 0.00 ^xy^	5.91 ± 0.03 ^zABC^	N.D.	81.90 ± 0.25 ^a^
	JUN	6.02 ± 0.04 ^yzAB^	4.91 ± 0.03 ^GH^	N.D.	70.91 ± 0.13 ^c^
	JUL	5.22 ± 0.02 ^DEFG^	4.96 ± 0.02 ^FGH^	N.D.	72.54 ± 0.24 ^b^
	AUG	6.36 ± 0.02 ^xy^	5.14 ± 0.04 ^EFGH^	N.D.	65.01 ± 0.39 ^e^
	SEP	5.39 ± 0.00 ^DEF^	9.54 ± 0.02 ^u^	N.D.	45.41 ± 0.17 ^j^
	OCT	4.76 ± 0.02 ^H^	8.23 ± 0.07 ^v^	N.D.	62.20 ± 0.22 ^f^
	NOV	3.86 ± 0.01 ^I^	6.75 ± 0.07 ^wx^	N.D.	66.42 ± 0.56 ^d^
	DEC	5.17 ± 0.01 ^EFGH^	9.12 ± 0.06 ^u^	N.D.	61.58 ± 0.12 ^g^
2021	JAN	3.68 ± 0.03 ^IJ^	6.23 ± 0.04 ^yz^	N.D.	47.21 ± 0.29 ^i^
	FEB	3.59 ± 0.03 ^IJ^	6.03 ± 0.04 ^yzAB^	N.D.	40.87 ± 0.30 ^m^
	MAR	3.52 ± 0.02 ^IJ^	6.79 ± 0.03 ^wx^	N.D.	41.60 ± 0.03 ^L^
	APR	3.06 ± 0.01 ^KL^	6.11 ± 0.04 ^yzA^	N.D.	48.11 ± 0.43 ^h^
	MAY	3.72 ± 0.02 ^IJ^	7.01 ± 0.02 ^w^	N.D.	42.78 ± 0.04 ^k^
	JUN	3.73 ± 0.01 ^IJ^	6.05 ± 0.06 ^yzAB^	N.D.	38.50 ± 0.05 ^p^
	JUL	3.36 ± 0.01 ^JK^	5.67 ± 0.05 ^ABCD^	N.D.	39.65 ± 0.10 ^o^
	AUG	3.66 ± 0.02 ^IJ^	5.91 ± 0.04 ^zABC^	N.D.	40.38 ± 0.22 ^n^
	SEP	3.64 ± 0.01 ^IJ^	5.88 ± 0.02 ^zABC^	N.D.	38.28 ± 0.12 ^p^
	OCT	2.86 ± 0.01 ^L^	5.47 ± 0.05 ^CDE^	N.D.	33.57 ± 0.07 ^t^
	NOV	2.81 ± 0.01 ^L^	5.28 ± 0.02 ^DEFG^	N.D.	36.13 ± 0.22 ^r^
	DEC	2.63 ± 0.02 ^L^	6.18 ± 0.01 ^yz^	N.D.	34.40 ± 0.15 ^s^

Within each morphotype table, values without a shared superscript letter differ significantly according to Tukey’s test (*p* < 0.05); N.D. = not detected. Letters refer to comparisons across collection months for the reported metabolite values.

**Table 6:** Quantification of 3D-anths in crude ethanolic extracts of morphotype AC3 collected monthly during 2020 and 2021.

Collection Year	Collection Month	Substance Content (mg/g Dry Extract) ± Standard Deviation (SD)			
		3′-Hydroxy- Carajurone	Carajurone	3′-Hydroxy- Carajurin	Carajurin
2020	JAN	2.66 ± 0.02 ^s^	1.57 ± 0.01 ^CD^	0.83 ± 0.00 ^N^	0.71 ± 0.00 ^O^
	FEB	9.10 ± 0.07 ^g^	2.80 ± 0.02 ^qr^	1.83 ± 0.01 ^y^	1.64 ± 0.01 ^BC^
	JUN	9.58 ± 0.06 ^f^	3.04 ± 0.04 ^lm^	1.78 ± 0.01 ^yz^	1.73 ± 0.02 ^zA^
	JUL	12.46 ± 0.04 ^c^	3.73 ± 0.02 ^j^	2.98 ± 0.01 ^mn^	2.20 ± 0.00 ^w^
	AUG	9.73 ± 0.05 ^e^	3.04 ± 0.02 ^lm^	1.79 ± 0.01 ^yz^	2.40 ± 0.02 ^v^
	SEP	7.96 ± 0.02 ^h^	2.86 ± 0.01 ^pq^	1.51 ± 0.00 ^D^	1.03 ± 0.00 ^M^
	OCT	13.93 ± 0.01 ^b^	4.02 ± 0.03 ^i^	3.02 ± 0.01 ^m^	1.67 ± 0.00 ^AB^
	NOV	12.28 ± 0.10 ^d^	3.31 ± 0.03 ^k^	2.59 ± 0.01 ^t^	1.35 ± 0.01 ^EF^
	DEC	14.07 ± 0.08 ^a^	3.78 ± 0.02 ^j^	2.94 ± 0.03 ^no^	1.60 ± 0.01 ^BC^
2021	JAN	2.88 ± 0.02 ^op^	1.24 ± 0.00 ^GHI^	0.64 ± 0.00 ^PQR^	0.44 ± 0.00 ^XYZ^
	FEB	3.04 ± 0.03 ^lm^	1.29 ± 0.01 ^FGH^	0.64 ± 0.00 ^PQR^	0.50 ± 0.00 ^VWX^
	MAR	2.84 ± 0.02 ^pq^	1.24 ± 0.00 ^GHI^	0.62 ± 0.00 ^PQRS^	0.54 ± 0.00 ^TUV^
	APR	3.09 ± 0.02 ^L^	1.31 ± 0.01 ^EFG^	0.66 ± 0.00 ^OP^	0.53 ± 0.00 ^UVW^
	MAY	3.28 ± 0.01 ^k^	1.36 ± 0.01 ^EF^	0.68 ± 0.00 ^OP^	0.52 ± 0.00 ^UVW^
	JUN	2.53 ± 0.02 ^tu^	1.14 ± 0.00 ^KL^	0.57 ± 0.00 ^RSTUV^	0.65 ± 0.00 ^OPQ^
	JUL	2.85 ± 0.02 ^pq^	1.18 ± 0.01 ^IJK^	0.58 ± 0.00 ^QRSTU^	0.41 ± 0.00 ^YZα^
	AUG	2.86 ± 0.01 ^pq^	1.22 ± 0.00 ^HIJ^	0.62 ± 0.00 ^PQRST^	0.37 ± 0.00 ^Zα^
	SEP	3.10 ± 0.03 ^L^	1.38 ± 0.00 ^E^	0.67 ± 0.00 ^OP^	0.41 ± 0.00 ^YZα^
	OCT	1.97 ± 0.00 ^x^	0.88 ± 0.00 ^N^	0.46 ± 0.00 ^WXY^	0.28 ± 0.00 ^β^
	NOV	2.50 ± 0.02 ^u^	1.08 ± 0.01 ^LM^	0.53 ± 0.00 ^UV^	0.36 ± 0.00 ^α^
	DEC	2.74 ± 0.01 ^r^	1.16 ± 0.00 ^JK^	0.56 ± 0.00 ^STUV^	0.34 ± 0.00 ^αβ^

Within each morphotype table, values without a shared superscript letter differ significantly according to Tukey’s test (*p* < 0.05);. Letters refer to comparisons across collection months for the reported metabolite values.

**Table 7:** Quantification of 3D-anths in crude ethanolic extracts of morphotype AC4 collected monthly during 2020 and 2021.

Collection Year	Collection Month	Substance Content (mg/g Dry Extract) ± Standard Deviation (SD)			
		3′-Hydroxy- Carajurone	Carajurone	3′-Hydroxy- Carajurin	Carajurin
2020	JAN	8.09 ± 0.08 ^g^	2.70 ± 0.02 ^w^	N.D.	N.D.
	FEB	10.88 ± 0.08 ^e^	2.82 ± 0.02 ^v^	0.63 ± 0.01 ^I^	0.36 ± 0.00 ^K^
	JUN	19.43 ± 0.06 ^a^	4.59 ± 0.06 ^s^	0.88 ± 0.01 ^H^	N.D.
	JUL	6.71 ± 0.06 ^k^	1.76 ± 0.00 ^z^	0.44 ± 0.00 ^JK^	N.D.
	AUG	9.01 ± 0.03 ^f^	2.30 ± 0.01 ^x^	0.54 ± 0.00 ^IJ^	N.D.
	SEP	6.00 ± 0.02 ^n^	1.88 ± 0.00 ^y^	N.D.	N.D.
	OCT	14.58 ± 0.08 ^b^	3.59 ± 0.01 ^t^	N.D.	1.78 ± 0.00 ^z^
	NOV	14.14 ± 0.09 ^c^	3.19 ± 0.02 ^u^	N.D.	0.50 ± 0.00 ^J^
	DEC	13.04 ± 0.10 ^d^	3.21 ± 0.03 ^u^	N.D.	1.04 ± 0.00 ^G^
2021	JAN	4.70 ± 0.05 ^r^	1.38 ± 0.01 ^CD^	N.D.	N.D.
	FEB	7.34 ± 0.06 ^i^	1.77 ± 0.00 ^z^	N.D.	N.D.
	MAR	5.07 ± 0.04 ^p^	1.27 ± 0.01 ^E^	N.D.	N.D.
	APR	4.96 ± 0.00 ^q^	1.29 ± 0.01 ^DE^	N.D.	N.D.
	MAY	6.54 ± 0.03 ^L^	1.63 ± 0.01 ^A^	N.D.	N.D.
	JUN	7.62 ± 0.04 ^h^	1.82 ± 0.01 ^yz^	N.D.	N.D.
	JUL	6.35 ± 0.04 ^m^	1.59 ± 0.00 ^A^	N.D.	N.D.
	AUG	6.71 ± 0.03 ^k^	1.56 ± 0.00 ^AB^	N.D.	N.D.
	SEP	7.23 ± 0.02 ^j^	1.78 ± 0.01 ^z^	N.D.	N.D.
	OCT	7.16 ± 0.02 ^j^	1.73 ± 0.00 ^z^	N.D.	N.D.
	NOV	5.78 ± 0.00 ^o^	1.48 ± 0.00 ^BC^	N.D.	N.D.
	DEC	4.58 ± 0.03 ^s^	1.15 ± 0.00 ^F^	N.D.	N.D.

Within each morphotype table, values without a shared superscript letter differ significantly according to Tukey’s test (*p* < 0.05); N.D. = not detected. Letters refer to comparisons across collection months for the reported metabolite values.

**Table 8:** Independent variables used in the experimental design for optimization of 3D-anths extraction from *Fridericia chica* leaves.

Independent Variables	Symbols	Codes		
		−1	0	1
Extraction time (min)	*X*1	15	30	45
Ethanol concentration * (%)	*X*2	70	80	100

* Ethanol concentration in ultrapure water.

**Table 9:** Experimental design for optimization of 3D-anths extraction from *Fridericia chica* leaves.

Assay	Codified Variables		Real Variables	
	*X*1	*X*2	Time (min)	Ethanol * (%)
A	−1	−1	15	70
B	−1	0	15	80
C	−1	1	15	100
D	0	−1	30	70
E	0	0	30	80
F	0	1	30	100
G	1	−1	45	70
H	1	0	45	80
I	1	1	45	100

* Ethanol concentration in ultrapure water.

## Data Availability

The data presented in this study are available within the article and Supplementary Materials.

## Supplementary Materials

The following supporting information can be downloaded at: https://www.mdpi.com/article/10.3390/ijms27188193/s1.
