# Supplementary material for: Bioactivity-Guided Optimization of 3-Deoxyanthocyanidin Extraction and Seasonal Mapping of Four Morphotypes of Fridericia chica (Bonpl.) L.G. Lohmann
Source: Int J Mol Sci. 2026 Sep 15;27(18):8193. doi: 10.3390/ijms27188193 (PMC13607019; doi:10.3390/ijms27188193)
Supplement: Supplementary file 1 [file ijms-27-08193-s001.zip › ijms-4520182-supplementary.pdf]

Supplementary Material

Table S1. Internal evaluation of the fitted models at the selected condition (30 min and 100% ethanol), based on the available 3 × 3 experimental-design data.

Response	Evaluation condition	Model-predicted value	Observed value at assay F	Reported standard deviation	Relative error (%)	Lower 95% prediction limit	Upper 95% prediction limit
Extract yield (%)	30 min; 100% ethanol	15.851	16.230	0.630	2.39	6.846	24.856
3′-Hydroxy-carajurone (% carajurin equivalents)	30 min; 100% ethanol	0.923	0.853	0.000*	7.60	0.731	1.115
Carajurone (% carajurin equivalents)	30 min; 100% ethanol	1.443	1.358	0.000*	5.88	1.161	1.724
Carajurin (%)	30 min; 100% ethanol	10.721	10.269	0.110	4.22	9.282	12.160
IC_{50}/72 h (µg/mL)	30 min; 100% ethanol	−14.201†	7.221	1.090	150.85	−81.644	53.242
CC_{50}/24 h (µg/mL)	30 min; 100% ethanol	−81.529†	53.760	1.170	165.94	−528.025	364.967
Selectivity index (SI)	30 min; 100% ethanol	8.822	7.500	NR‡	14.99	−3.526	21.171

Notes: Models were refitted from the nine supplied conditions as $y = b0 + b1$ (time) + $b2$ (time^2) + $b3$ (ethanol concentration) + $b4$ (ethanol concentration^2), consistent with residual df = 4 and the R^2 values in the supplied effect-estimate output. Relative error (%) = |observed − predicted| / |predicted| × 100. Prediction intervals were calculated for a future observation at 30 min and 100% ethanol using t 0.975,4 and the model residual mean square. Reported standard deviations were taken from the corresponding assay-F results available in the revised manuscript. *Values reported as 0.00 after rounding; additional decimal places were unavailable. †Negative fitted concentrations are physically inadmissible and demonstrate poor predictive suitability of the unconstrained polynomial models for IC_{50} and CC_{50}. ‡NR, not reported; SI variability cannot be reconstructed reliably without paired raw IC_{50} and CC_{50} replicate data.

Table S2. Analysis of the Pearson correlation among 3D-anth and the climatic factors evaluated for AC1 morphotype

3D-anth	Average temperature (°C)	Average humidity (%)	Dew point (°C)	Pressure (hPa)	Wind speed (m/s)	Radiation (kJ/m²)	Accumulated precipitation (mm)
3′-hydroxy-Carajurone	0.055	-0.429	-0.091	0.194	0.131	-0.073	0.170
Carajurone	0.160	-0.407	0.029	0.076	0.188	-0.016	0.213
3′-hydroxy-Carajurin	-0.365	0.228	-0.310	0.250	-0.188	-0.130	-0.319
Carajurin	0.072	-0.461*	-0.088	0.197	0.167	-0.060	0.149

*Significant at $p < 0.05$.

Table S3. Analysis of the Pearson correlation among 3D-anth and the climatic factors evaluated for AC2 morphotype

3D-anth	Average temperature (°C)	Average humidity (%)	Dew point (°C)	Pressure (hPa)	Wind speed (m/s)	Radiation (kJ/m²)	Accumulated precipitation (mm)
3′-hydroxy-Carajurone	-0.208	-0.444*	-0.371	0.392	0.180	0.214	-0.400
Carajurone	0.331	0.193	0.411	-0.262	0.109	0.253	0.274
3′-hydroxy-Carajurin	ND	ND	ND	ND	ND	ND	ND
Carajurin	0.111	-0.269	0.025	0.078	-0.012	0.008	0.298

*Significant at $p < 0.05$; ND = not detected.

Table S4. Analysis of the Pearson correlation among 3D-anth and the climatic factors evaluated for AC3 morphotype

3D-anth	Average temperature (°C)	Average humidity (%)	Dew point (°C)	Pressure (hPa)	Wind speed (m/s)	Radiation (kJ/m²)	Accumulated precipitation (mm)
3′-hydroxy-Carajurone	0.089	-0.144	0.054	0.027	0.093	-0.008	0.276
Carajurone	0.104	-0.206	0.047	0.039	0.130	0.029	0.240
3′-hydroxy-Carajurin	0.096	-0.117	0.071	-0.002	0.062	-0.024	0.262
Carajurin	-0.019	-0.257	-0.101	0.186	0.038	-0.031	0.164

Table S5. Analysis of the Pearson correlation among 3D-anth and the climatic factors evaluated for AC4 morphotype

3D-anth	Average temperature (°C)	Average humidity (%)	Dew point (°C)	Pressure (hPa)	Wind speed (m/s)	Radiation (kJ/m²)	Accumulated precipitation (mm)
3′-hydroxy-Carajurone	0.123	-0.292	0.040	0.006	0.076	-0.005	0.066
Carajurone	0.204	-0.333	0.110	-0.056	0.125	0.041	0.091
3′-hydroxy-Carajurin	-0.039	-0.493*	-0.213	0.261	0.042	-0.102	0.017
Carajurin	0.224	0.121	0.290	-0.256	0.078	0.037	0.357

*Significant at $p < 0.05$.
